# Model Test on the Multi-Effects of Tunneling and Excavation upon an Existing Pile in Clay

**DOI:** 10.3390/ma19163453

**Published:** 2026-08-14

**Authors:** Hongguo Diao, Zhiwei Zhou, Qiang Li, Haibo Hu, Xinquan Wang, Gang Wei, Hanmei Luo, Yuhao Lu

**Affiliations:** 1 School of Engineering, Hangzhou City University, Hangzhou 310015, China; diaohg@hzcu.edu.cn (H.D.); ziv_zhou@foxmail.com (Z.Z.); wangxq@hzcu.edu.cn (X.W.); hzcuweig@163.com (G.W.); mayluo0115@gmail.com (H.L.); 2 College of Civil Engineering and Architecture, Zhejiang University, Hangzhou 310058, China; 22512225@zju.edu.cn; 3 PowerChina Huadong Engineering (Shenzhen) Corporation Limited, Shenzhen 518100, China; qiangli1991@outlook.com

**Keywords:** excavation unloading, existing pile foundation, additional response, model test

## Abstract

Existing studies often analyze tunnels and excavations as isolated projects, but in practice they are often constructed sequentially or simultaneously. This paper investigates their combined effects on adjacent pile foundations in clay using constant-gravity model tests and 3D numerical methods, considering time-dependent clay behavior and small-strain stiffness. Two combined construction sequences of tunnelling and excavation adjacent to an existing pile were investigated: (i) tunnel excavation followed by basement excavation (i.e., test TEP); (ii) basement excavation followed by tunnel excavation (i.e., test ETP). Results show that construction sequence has little effect on surface settlement but significantly influences pile response. Pile top settlement increases considerably when tunnelling occurs near piles. The TEP sequence reduces cumulative pile top settlement by 0.38% of the pile diameter and reduces the maximum cumulative bending moment by approximately 31% compared with the ETP sequence. Additionally, tunnel and excavation construction generate negative excess pore water pressure in the surrounding soil, which dissipates over time. The pressure increases with proximity to the tunnel face or excavation base.

## 1. Introduction

With the deepening of urbanization, the development and utilization of underground space is becoming increasingly widespread and intensive. Tunnel and excavation, as two typical forms of underground engineering, are extremely common in the field of geotechnical engineering. The original stress balance of the strata is disrupted by excavation unloading, inevitably leading to stress redistribution and deformation within the affected soil mass [1]. The resulting soil displacement acts on the pile foundations of adjacent existing structures, inducing additional deformation and internal forces in the piles, which, in severe cases, may endanger the safety and stability of the superstructure [2,3]. As urban underground space development becomes more intensive and networked, the distance between new underground structures and existing pile foundations is constantly decreasing, making it crucial to accurately assess the impact of excavation unloading on the performance of adjacent piles [4]. Existing research on the effects of excavation unloading on adjacent piles has been primarily confined to two scenarios: tunneling alone or excavation alone, both of which are classified as classic passive pile problems. Field measurements, theoretical analysis, numerical simulation, and physical model tests are the primary methods employed.

Field evidence concerning tunneling effects remains comparatively scarce because installing instruments is difficult; nevertheless, the available observations consistently document additional settlement and lateral movement of nearby pile foundations [5,6,7,8,9,10]. In an early study published in 1986, Attewell et al. [5] examined a tunnel driven laterally past a pile-supported structure and reported that coating the pile shaft with asphalt could lessen tunnelling-induced negative skin friction. Later London case studies involved a tunnel passing pile-supported buildings in stiff clay at a minimum horizontal separation of approximately 1 m. With a tunnelling volume loss of 2%, the recorded maximum horizontal displacement of the piles reached 10 mm [6,7]. Subsequent work showed that the induced pile response depends on tunnel-pile geometry, including transverse separation and the longitudinal distance from the tunnel face to the pile [8,9,10]. Analytical and numerical techniques provide important means of studying these interactions. Such analyses are commonly formulated using either a global approach or a two-stage approach. In global analyses, numerical procedures such as the finite element method (FEM) and finite difference method (FDM) simulate excavation while representing the pile and surrounding ground as a coupled system [11,12,13]. A two-stage analysis first estimates free-field ground movement due to tunnelling in the absence of the pile; those movements are then imposed on the pile through a boundary element or load-transfer formulation to obtain its additional deformation and internal forces [14,15,16]. Tunnel-induced shear strains in the adjacent ground, however, generally fall within the small-strain interval of 0.01–1% [17]. Soil stiffness also varies with both stress path [18] and strain level [19]. Consequently, reliable prediction of pile response requires a constitutive model that can reproduce stiffness degradation at small strains. Physical model studies have likewise clarified tunnel-pile interaction. Morton and King [20] reported that tunnelling through soft soil substantially influenced pile bearing capacity and settlement. For a single tunnel driven near a pile toe in dense sand, Ghahremannejad et al. [21] measured the largest pile bending moment at roughly 1.5 Dt above the springline and found that it increased as volume loss rose. Meguid and Mattar [22] studied a single tunnel interacting with a pile group in clay and observed decreasing tunnel-lining bending moments as pile-tunnel spacing was reduced below 1 Dt.

Regarding excavation impacts, field observations have confirmed that soil deformation outside the excavation is induced by construction activities, thereby causing displacement of adjacent pile foundations. For instance, Goh et al. [23] recorded maximum horizontal pile displacements reaching 2.8% of the pile diameter, while Ou et al. [24] delineated the primary influence zone of excavation-induced surface settlement. Notably, Zheng et al. [25] observed that even when retaining wall deformation was constrained to within 0.11% of the excavation depth, maximum horizontal displacements of adjacent short piles still reached 13.2 mm. However, with real-time grouting compensation, pile displacements were reduced by nearly 50%, demonstrating that active compensation for soil stress loss ahead of the piles can effectively mitigate unloading disturbances. In physical modeling, Ng et al. [26] revealed that supported excavations in sands induce downward load transfer and significant additional settlement in adjacent friction piles. Furthermore, systematic investigations by Leung and colleagues [27,28,29] have addressed the effects of unsupported excavations on end-bearing piles and pile groups in both sands and clays. Results from clay tests proved particularly critical, highlighting the time-dependent nature of excavation unloading: pile responses were observed to evolve continuously post-excavation due to excess pore water pressure dissipation. Moreover, this time effect was found to intensify with greater excavation depths or reduced support system stability [28,29]. Theoretically, the two-stage analysis method has been widely adopted [30,31,32], while numerically, the influences of factors such as excavation geometry, relative position, support stiffness, soil stress history, and pile head constraints have been extensively explored [33,34,35]. Using small-strain hardening soil and hypoplastic models, Shakeel and Ng [34] and Soomro et al. [35] simulated excavation effects on adjacent pile groups in clay, consistently finding that continuous settlement is driven by excess pore water pressure dissipation. Analogous to tunneling, shear strains in the surrounding soil induced by excavation are predominantly confined to the small-strain range. Consequently, the adoption of constitutive models capable of capturing small-strain stiffness characteristics is deemed essential for accurately analyzing excavation effects on pile foundations.

Even for identical excavation configurations, such as twin tunnels, variations in excavation sequence have been shown to induce distinct pile foundation responses. Field monitoring [36], centrifuge tests [37,38], and numerical simulations [39,40] consistently demonstrate that the response induced by subsequent excavation is not linearly superimposable. This non-linearity is attributed to alterations in the soil stress state and pile load redistribution triggered by the prior excavation. Given that tunnels and excavations represent distinct construction forms inducing divergent soil stress paths, and considering the stress-path dependence of soil stiffness, the excavation sequence is expected to critically influence the final pile response in complex combined tunnel-excavation scenarios [41,42,43,44]. Consequently, the underlying mechanisms warrant further investigation.

Overall, although pile responses under single excavation methods have been extensively studied, the mechanisms of combined tunnel and excavation unloading on pile foundations remain poorly understood. Moreover, due to the complexity of soil behavior, this problem cannot be simply treated as a superposition of the two individual cases. Homogeneous dry sand or remolded kaolin were adopted in most physical model tests, which differ substantially from natural undisturbed soils in stress history, inherent structure and anisotropy [45,46,47]. The issue is particularly complex in clay, involving time effects dominated by excess pore water pressure dissipation, the influence of stress path changes during unloading, and the need to accurately characterize soil stiffness within the small-strain range. However, the response mechanisms and long-term evolution of adjacent piles under combined unloading have not yet been systematically revealed by existing studies, representing a significant gap in current research. Dynamic pile tests are generally performed under two main conditions: (i) during or immediately after pile installation, to check pile integrity and detect construction defects; and (ii) at the project site before superstructure construction, to evaluate bearing capacity and load–settlement behavior when conventional static load tests are difficult to conduct due to site limitations, tight schedules, or high costs.

Therefore, this research examines the effects of the sequence of combined tunnel-excavation unloading on the performance of adjacent single piles in clay strata. By conducting 1 g scaled model tests combined with numerical simulations, this paper investigates the evolution of additional deformation, internal force distribution, and load transfer mechanisms of pile foundations under different excavation sequences. The findings aim to provide a theoretical reference and basis for the scientific design and safe construction of similar projects.

## 2. Indoor Model Test

### 2.1. Test Program

The experimental matrix for the constant-gravity indoor scaled model tests is summarized in the Table 1 below. The abbreviations T, E, and P denote Tunnel, Excavation, and single Pile, respectively. Taking the TEP case as an example, this sequence signifies an investigation into the effects on an adjacent single pile induced by tunnel excavation followed by excavation.

**Figure 1:**
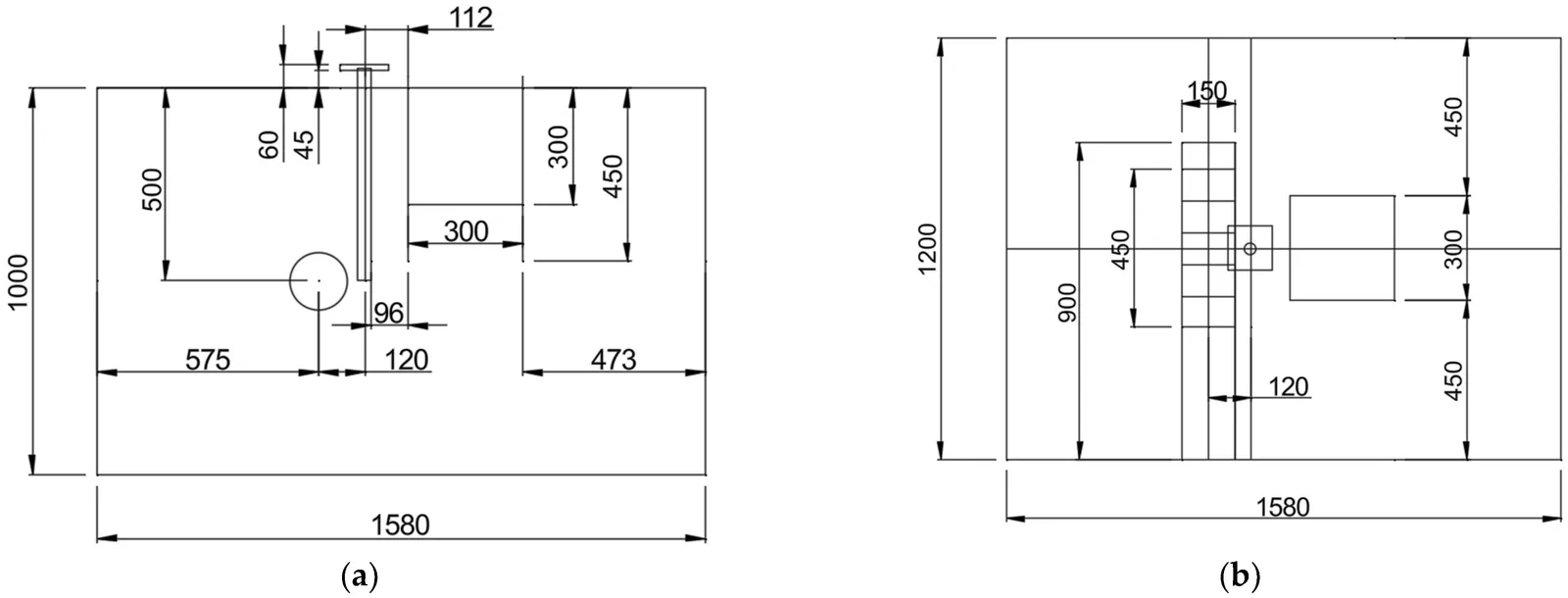
Scaled model test: (**a**) Elevation view of pile. (**b**) Plan view of pile.

Figure 1 presents the cross-sectional and plan views of the constant-gravity indoor scaled model test for a single pile. The dimensions of the model box were configured as 1580 mm in length, 1200 mm in width, and 1000 mm in height. A geometric scaling factor of 1:40 was adopted, corresponding to prototype dimensions of 63.2 m in length, 48 m in width, and 40 m in depth.

In this study, a 1 g constant-gravity physical model was employed with a geometric scaling ratio C_L_ = 1/40. Field-retrieved undisturbed intact clay served as the model soil, ensuring equivalent soil density and gravitational acceleration between the model and prototype, from which the fundamental similitude relationships were established. The scaling factors for stress, excess pore water pressure, permeability, ground displacement, and consolidation time are all identical to the geometric scale C_L_ = 1/40, implying that the model values of these parameters amount to 1/40 of their prototype counterparts. For force-based quantities such as concentrated pile-head loads and axial forces along the shaft, the scaling factor becomes C_L_^3^ = 1/64,000, whereas the scaling factor for pile bending moments follows C_L_^4^ = 1/2,560,000. Given that strain is dimensionless, its scaling factor is unity, allowing strain measurements from the model to be directly extrapolated to the prototype without any conversion.

### 2.2. Test Materials and Equipment

#### 2.2.1. Test Soil Materials

Granular materials are frequently employed as test soils in geotechnical experiments; nevertheless, the majority of studies utilize sand or clay owing to the complexity of granular materials [48,49,50,51]. The clay utilized in this model test was sourced from Section SG12-8 of the civil works for Phase I of Hangzhou Metro Line 12, Zhejiang Province. The soil is characterized by a grayish-black appearance, high initial moisture content, and significant cohesion. The raw soil was air-dried, pulverized, and sieved through a 2-mm sieve to prepare the dry soil for testing. Using the combined liquid-plastic limit method, the liquid limit, plastic limit, and plasticity index were determined to be 40.75%, 18.46%, and 22.29%, respectively.

#### 2.2.2. Model Box and Control System

A geometric scale ratio of 1:40 was adopted. The model box was constructed with net dimensions of 1580 mm in length, 1200 mm in width, and 1270 mm in height. To facilitate visual observation, the front panel was fabricated from organic glass (PMMA), while the remaining side walls were constructed of 10-mm-thick steel plates. Steel stiffeners were installed on the left, right, and rear walls to ensure overall structural rigidity. Sliding rails allowing longitudinal movement were mounted on the model box to support an instrumentation frame, onto which the required displacement sensors were fixed.

**Figure 2:**
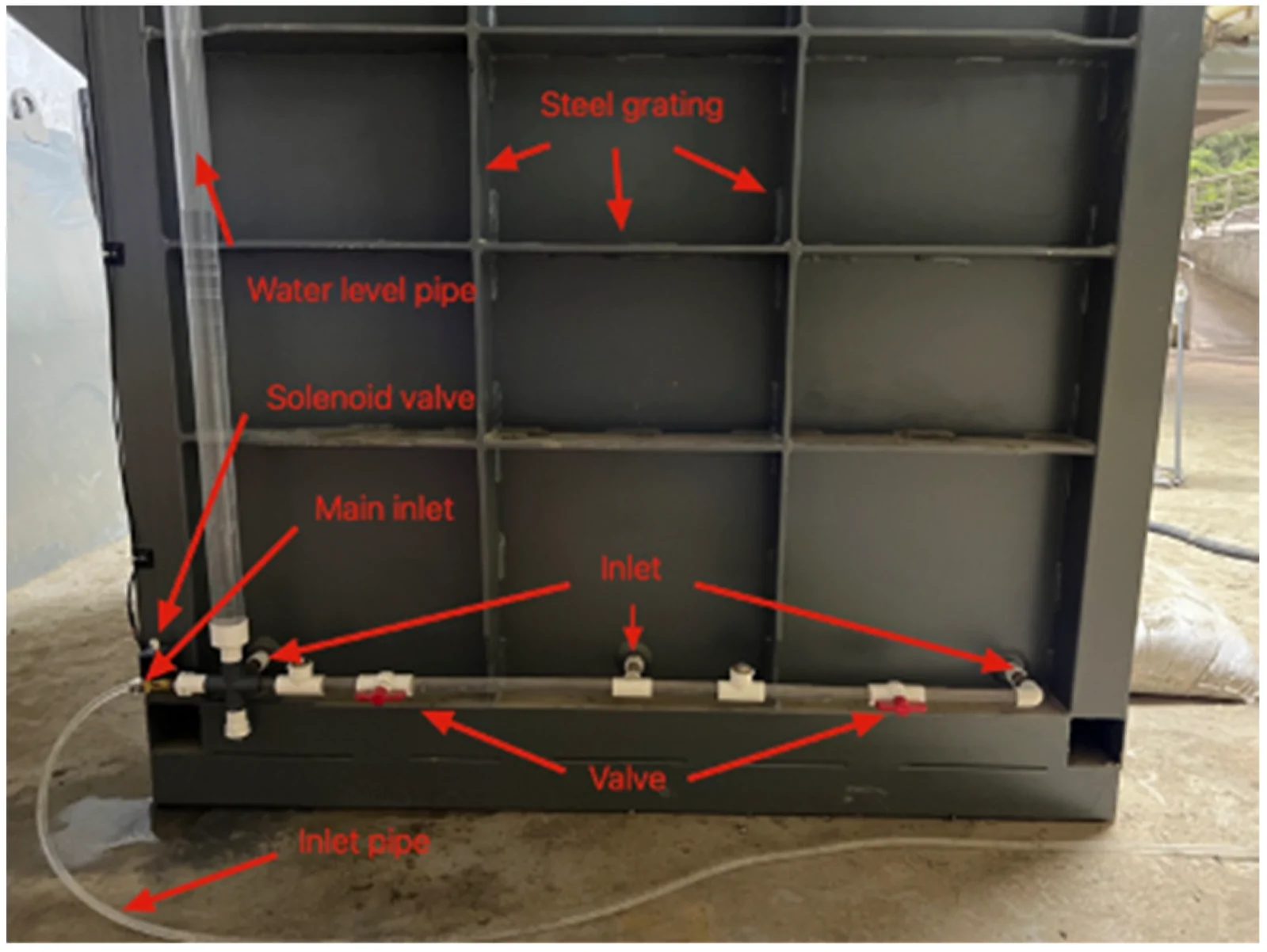
Water inlet system.

**Figure 3:**
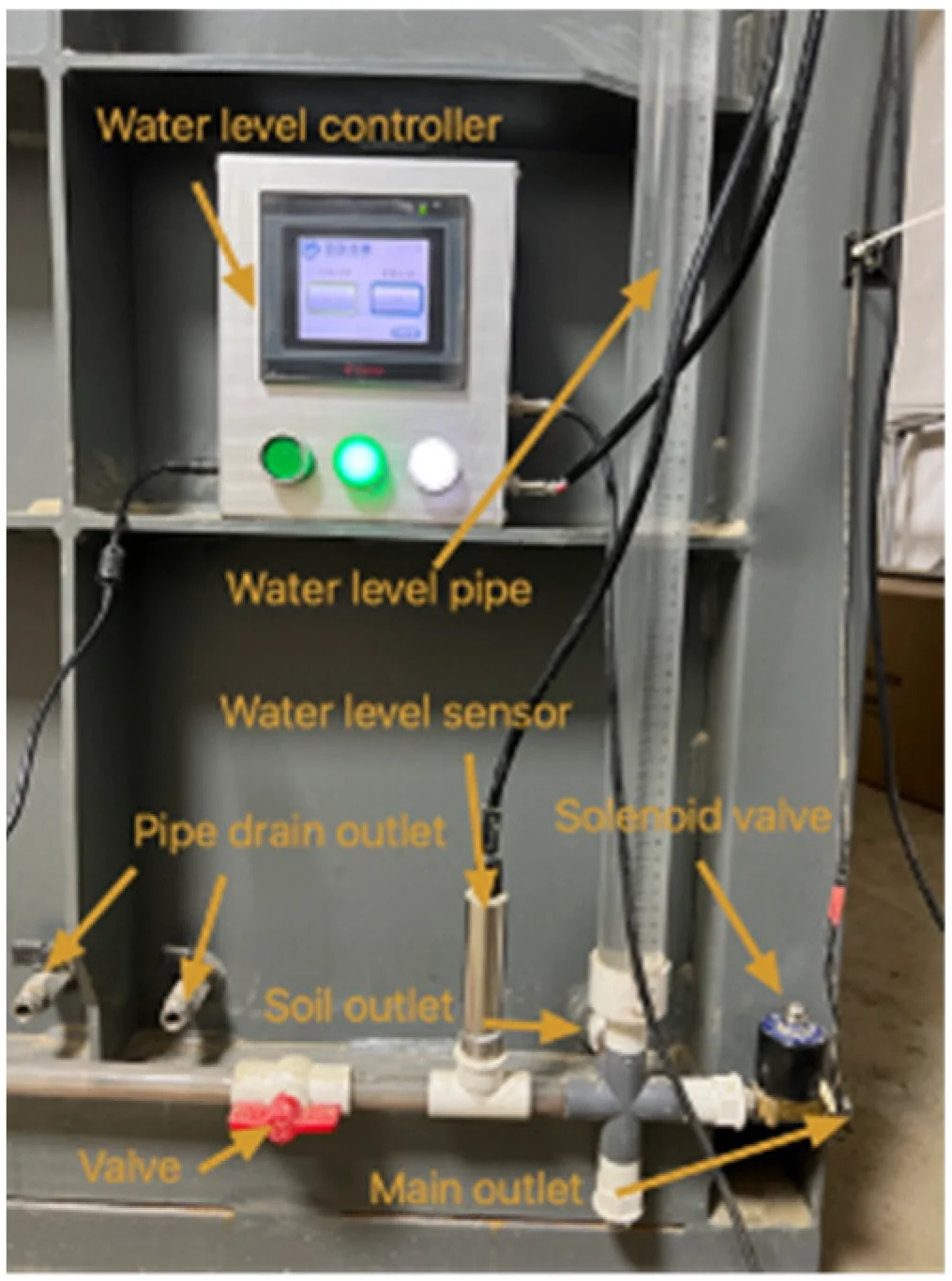
Drainage system.

The groundwater control system comprises a water level controller, sensors, solenoid valves, observation tubes, a main inlet, and a main outlet. As illustrated in Figure 2 and Figure 3, the water level within the model box can be regulated to any desired elevation by the controller. Water level sensors are employed to monitor the water status and transmit electrical signals to the controller. Solenoid valves were installed at both the main inlet and outlet to regulate water inflow and outflow, respectively. Consequently, the water level can be visually monitored via the observation tubes and simultaneously displayed on the controller’s screen.

#### 2.2.3. Model Components

(1) Model pile

**Figure 4:**
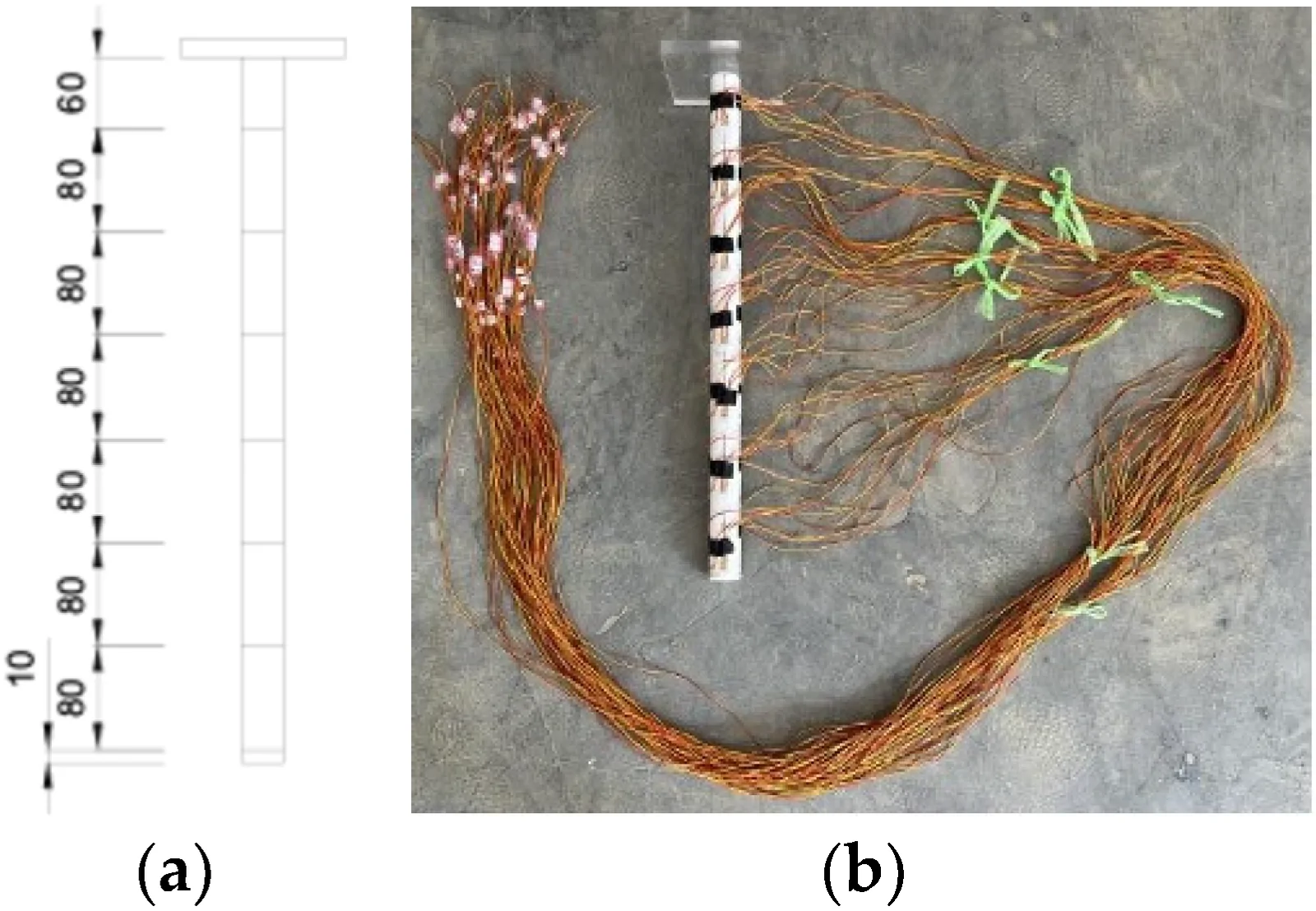
Single-pile model: (**a**) schematic diagram; (**b**) actual product image.

A friction pile was investigated in this study, wherein the majority of the vertical load at the pile head is sustained by shaft resistance. The model pile was fabricated from rigid PVC pipe, featuring an outer diameter of 32 mm, an inner diameter of 28 mm, and an embedment depth of 500 mm. The pipe bottom was sealed with a cap and coated with waterproof adhesive to prevent infiltration of pore water from the clay into the pipe. Based on the 1:40 geometric scale, the model simulates a prototype pile with a diameter of 1.28 m and a length of 20 m. BX120-3AA resistance strain gauges were bonded at equal intervals along the pile shaft. The pile cap was constructed from an organic glass (PMMA) plate measuring 128 mm × 128 mm × 15 mm (Figure 4). A circular groove, 32 mm in diameter and 5 mm in depth, was machined at the center of the cap.

(2) Model tunnel

**Figure 5:**
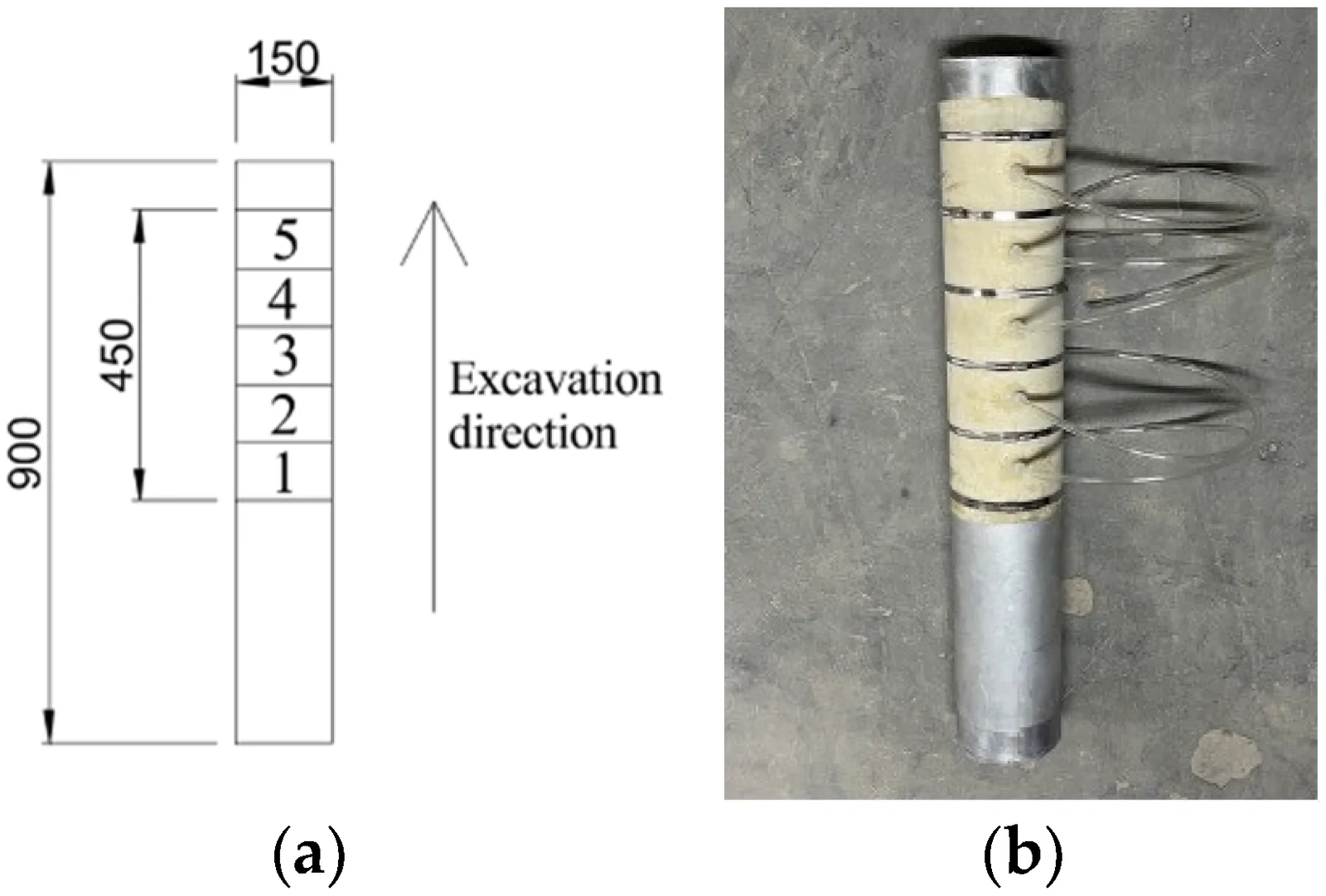
Tunnel model: (**a**) schematic diagram; (**b**) actual product image.

The tunnel model employed in this study is illustrated in Figure 5. In existing model tests, employing the volume loss rate as a control parameter to simulate soil disturbance during tunneling has been established as the most prevalent and accepted methodology. This approach was adopted herein, wherein volume loss was simulated by draining liquid from the latex membrane. A target volume loss rate of 5% was applied. The model assembly comprised an aluminum alloy tube, latex membranes, circular end plates, clamping rings, suction cups, and PU tubing. The aluminum alloy tube served as the tunnel lining, measuring 900 mm in length and 150 mm in diameter. The simulated excavation zone spanned 450 mm and was divided into five segments, each 90 mm in length. Both ends were sealed with 150-mm-diameter circular aluminum plates and coated with waterproof adhesive to prevent water infiltration from the clay. The latex membrane was segmented by clamping rings, with each segment individually connected to a PU tube. This configuration enabled independent injection and drainage of liquid for each section. PU tubing with a 10-mm outer diameter was matched to the drainage port; one end was connected to the port for fluid discharge, while the other was attached to each latex segment via a suction cup.

Under the 1:40 geometric scale, the model tunnel (150 mm in diameter, 450 mm in excavation length) corresponds to a prototype tunnel of 6.0 m diameter and 18.0 m excavation length. The distances from the tunnel centerline to the model box boundaries exceed 4.5 times the tunnel diameter, a margin deemed adequate to suppress boundary interference with the measured responses.

(3) Model excavation

**Figure 6:**
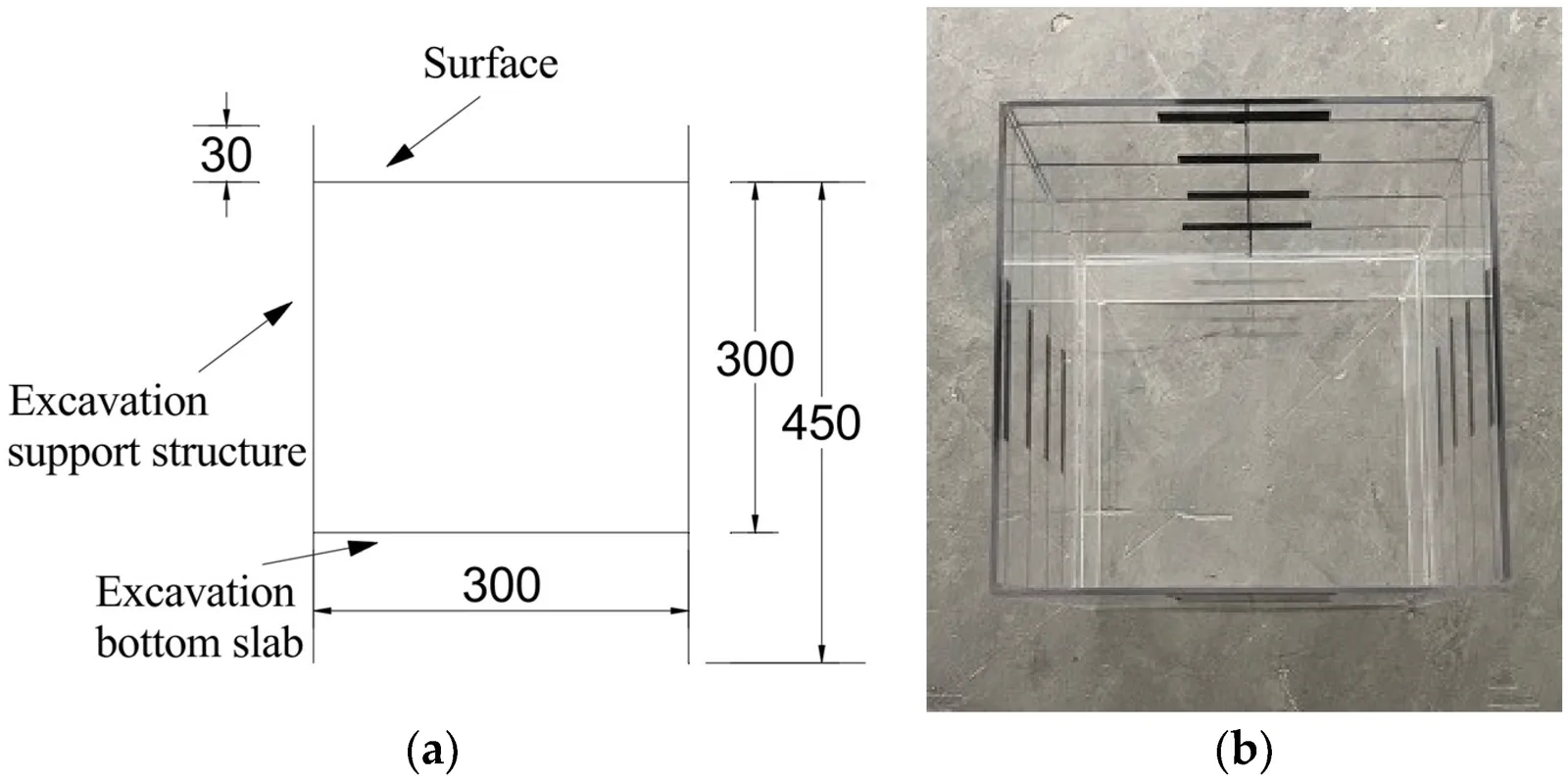
Excavation model: (**a**) schematic diagram; (**b**) actual product image.

The excavation model employed in this study is illustrated in Figure 6. The model was assembled from four 4-mm-thick organic glass (PMMA) panels. A base plate was installed at a depth of 300 mm below the ground surface to prevent upward seepage of pore water from the underlying clay. The model dimensions were 300 mm (length) × 300 mm (width) × 480 mm (height), configured with an embedment depth of 450 mm and an excavation depth of 300 mm; the insertion ratio was specified as 0.5. The retaining structure extended 30 mm above the ground surface to prevent soil intrusion into the ZnCl_2_ heavy fluid contained within the excavation. The elastic modulus of the organic glass panels was taken as 3100 MPa. Based on the scaling law for flexural rigidity, the model was designed to be equivalent to a KSP-IIIA steel sheet pile wall. The calculated flexural stiffness per unit width was approximately 42.325 MN·m^2^/m.

Under the 1:40 scale, the model excavation (300 mm × 300 mm in plan, 300 mm deep) represents a prototype excavation of 12.0 m × 12.0 m in plan and 12.0 m in depth. The clearance between the excavation support structure and the nearest model box wall was maintained at over 3 times the excavation depth to mitigate boundary effects.

#### 2.2.4. Data Acquisition System

**Figure 7:**
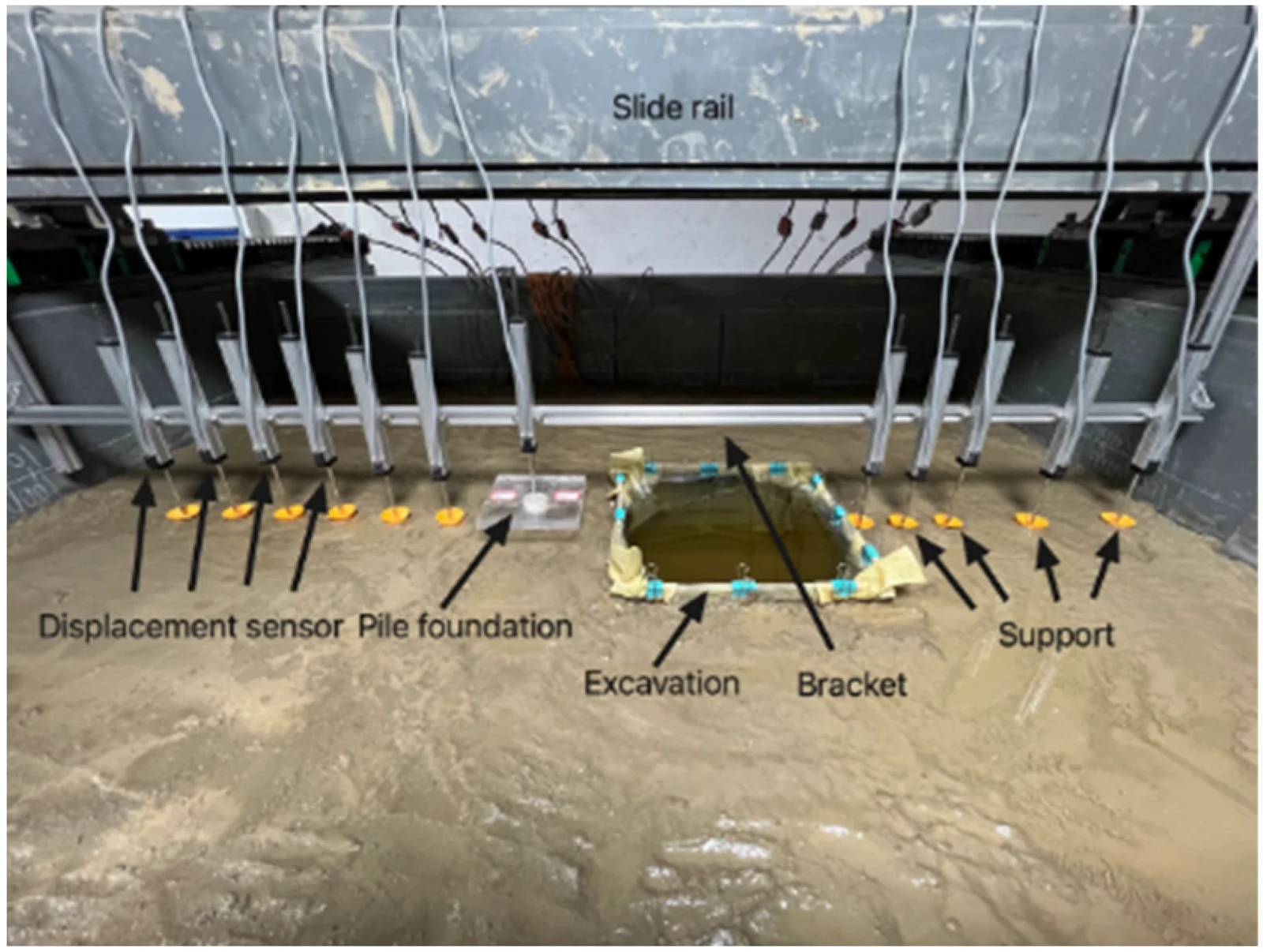
Measurement of pile top displacement and ground surface settlement.

**Figure 8:**
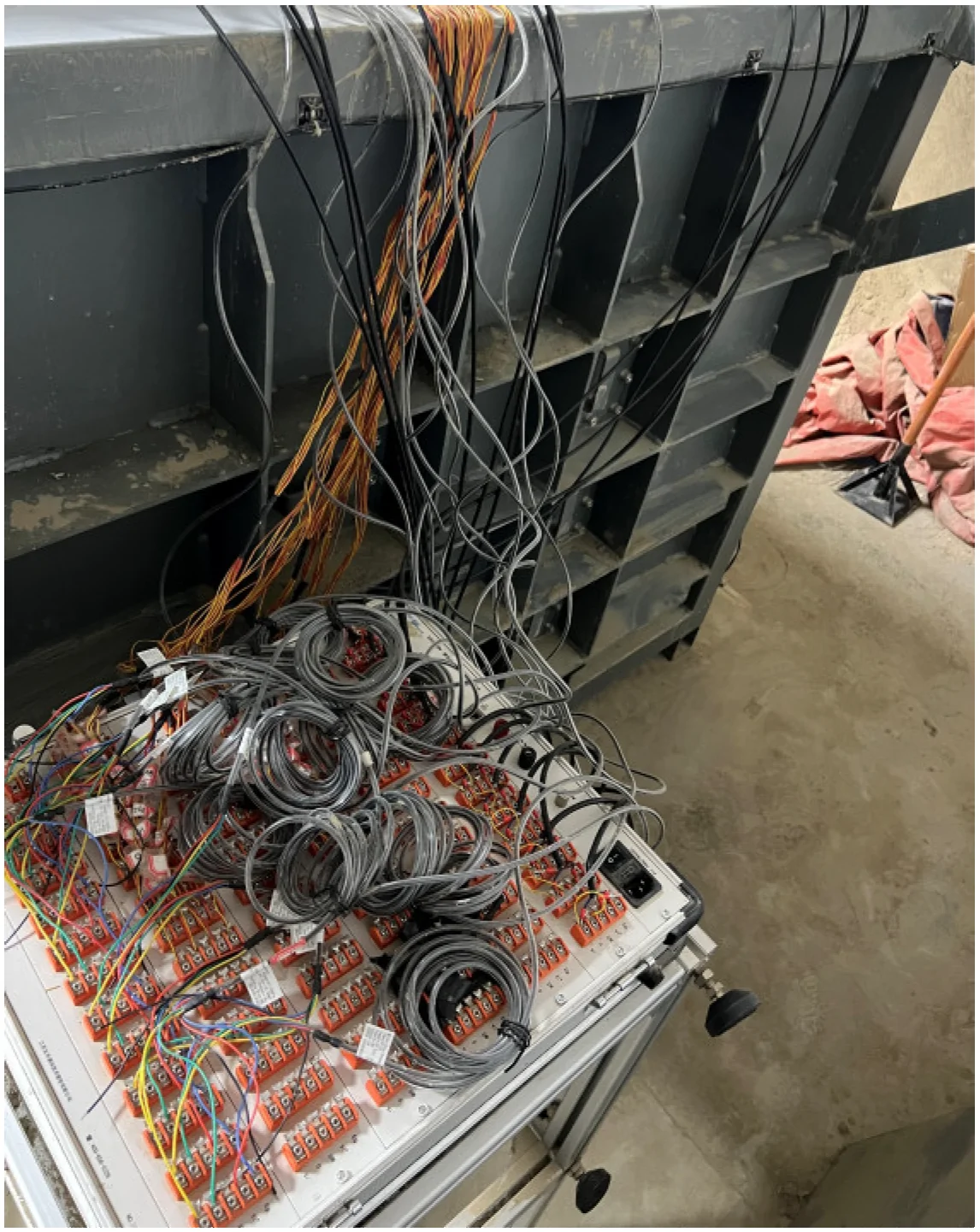
Stress–strain testing system.

This model simultaneously collected data on pile top displacement, surface settlement, pile axial force, pile bending moment, and pore water pressure. Pile top displacement and surface settlement were measured using a KTR3 linear displacement sensor (Shenzhen Miran Technology Co., Ltd., Shenzhen, China) with a range of 100 mm, mounted on a horizontal support via a hardware bracket: the probe was placed at the center of the pile cap to measure pile top displacement, and at the center of the surface settlement support to measure surface settlement (Figure 7). Pile axial force and bending moment were measured using BX120-3AA resistance strain gauges (Shenzhen Taida Century Technology Co., Ltd., Shenzhen, China) (resistance 120 ± 0.3 Ω, sensitivity coefficient 2.111%, wire size 3.0 × 2.5 mm). After the strain gauges were connected in a full-bridge configuration and calibrated, they were connected to a Donghua DH3816N data acquisition instrument (Donghua Test, Jingjiang, China) (72 channels) and recorded using DHDAS software (V7.2) (Figure 8). Pore water pressure was measured using GT-25 miniature sensors (Shenzhen Norwis Technology Co., Ltd., Shenzhen, China) (range 20 kPa, diameter 10 mm, length 10 mm, accuracy 0.2% FS). A total of 12 sensors were deployed around the tunnel, excavation, and pile foundation to analyze the dissipation of excess pore water pressure and its impact on the pile foundation response.

### 2.3. Test Procedure

(1) Additionally, 27 L of ZnCl2 solution was prepared with a unit weight matching that of the soil sampled during the single-pile static load test, to simulate excavation via fluid displacement. Cotton plugs were inserted into the three inlet and outlet ports to prevent sand intrusion into the piping system. Reference lines were marked on the internal steel sidewalls to facilitate positioning. The tunnel, pile, and excavation models were precisely aligned and marked using aluminum alloy square tubes and taut wires. Finally, geotextiles were adhered to the sidewalls to accelerate water infiltration during saturation.
(2) First, a 100-mm-thick sand layer was deposited and compacted using a tamper. Subsequently, clay sieved through a 2 mm mesh was mixed with water to achieve a moisture content of 18% and cured for over 24 h. The prepared clay was then placed in layers atop the sand, with each 50 mm layer compacted until the target height of 1000 mm was reached. The tunnel, pile, and excavation models were installed at their designated positions during the compaction process.
(3) The water level was set to 1000 mm (flush with the ground surface) using the calibrated controller. The inlet valve was opened to fill the box to the target level. After standing for 24 h, the system was refilled to restore the water level. Displacement sensors were then installed to monitor surface settlement. The subsequent procedure was initiated only after the settlement rate stabilized below 0.01 mm/h.
(4) Strain gauges and micro-pore water pressure sensors were connected via channels consistent with those used during calibration. Initial readings for displacement and pile axial force were zeroed. Graded loading was applied to the pile cap. Upon stabilization of pile head settlement, displacement and bending moment readings were reset, whereas axial force and pore water pressure data were retained.
(5) Tunnel excavation was simulated by opening the external PU tube valves. Effluent was collected in graduated cylinders to ensure volume balance between injection and discharge. Upon stabilization of surface and pile head settlements induced by the first segment, the remaining four segments were excavated sequentially. Following data stabilization, excavation simulation was commenced by opening the valves connected to the excavation model. Heavy ZnCl2 fluid was discharged in increments of 6.75 L and collected in large graduated cylinders until the excavation was completed.
(6) Upon completion of data acquisition, all data were exported. The instrumentation was dismantled, and the soil along with the tunnel, pile, and excavation models were removed from the box. The model box was subsequently cleaned, marking the conclusion of the sequential tunnel-then-excavation test.

## 3. Analysis of Experimental Results

### 3.1. Determination of the Ultimate Bearing Capacity of Single Pile

**Figure 9:**
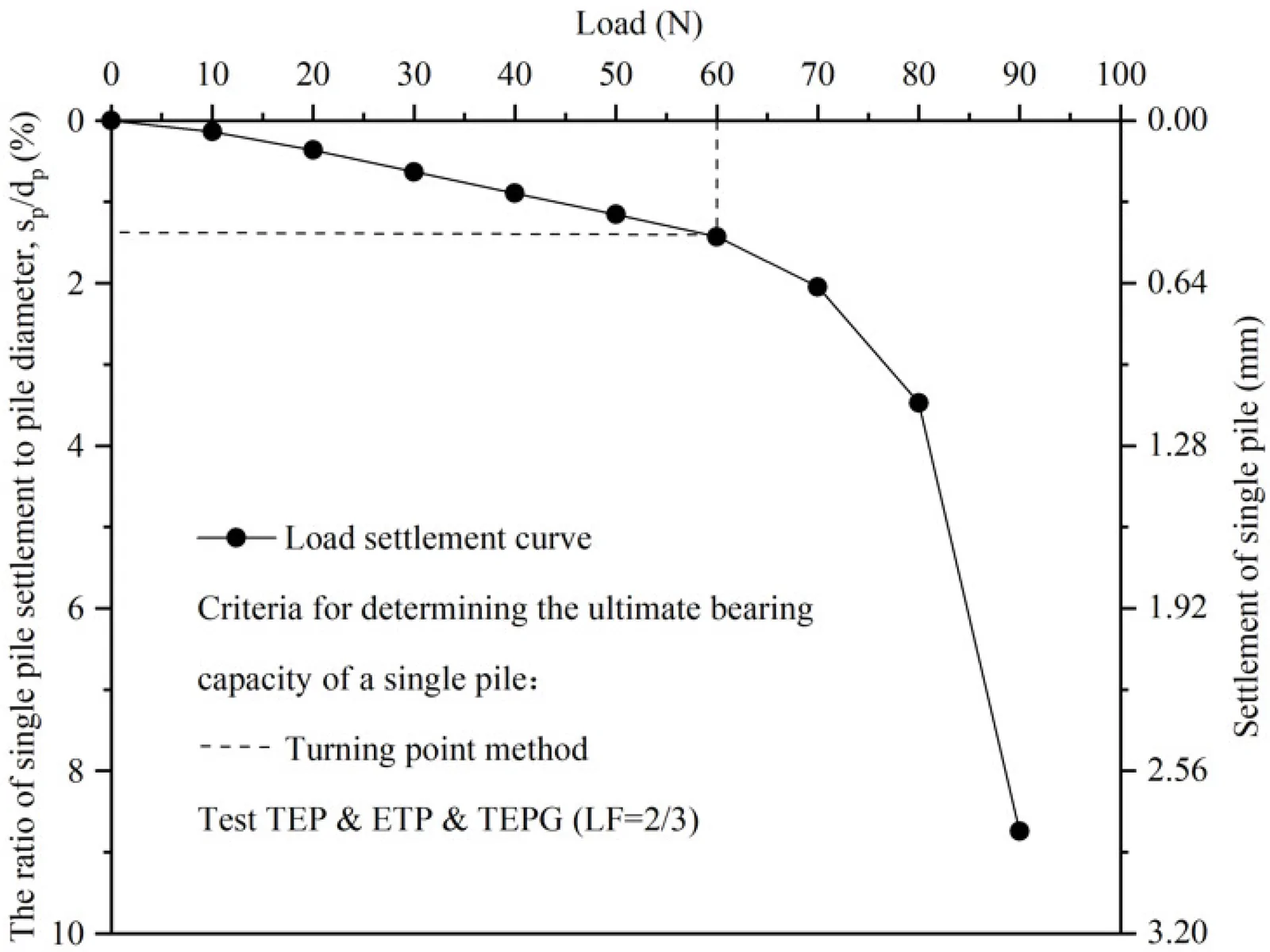
Load settlement curve of single pile.

To determine the working load at the pile top in the experiment, a static load test on a single pile was conducted under identical conditions (no tunnel or excavation) to determine the ultimate bearing capacity of the model pile. During the test, the working load at the pile top was gradually increased to 90 N using weights. A linear displacement sensor was used to measure the pile top settlement, resulting in the single-pile load-settlement curve shown in Figure 9. For single pile with a steeply sloping load-settlement curve, the ultimate bearing capacity is often taken as the load corresponding to the inflection point. Therefore, the ultimate bearing capacity of the single pile is 60 N. The pile top loading coefficient is taken as 2/3; hence, the pile top load for both the TEP and ETP tests is 40 N.

### 3.2. Surface Deformation

#### 3.2.1. Surface Deformation Caused by Tunnel Excavation

**Figure 10:**
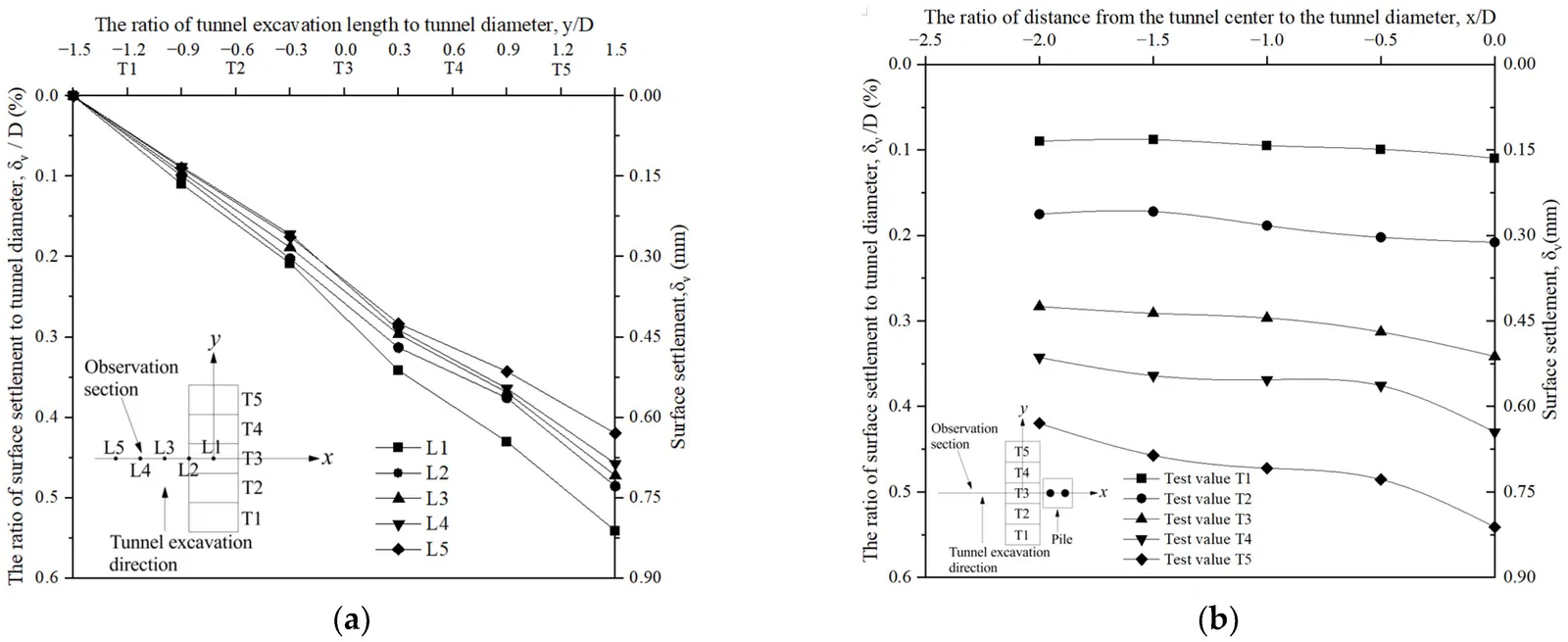
Net surface settlement caused by tunnel excavation in test TEP: (**a**) y/D; (**b**) x/D.

**Figure 11:**
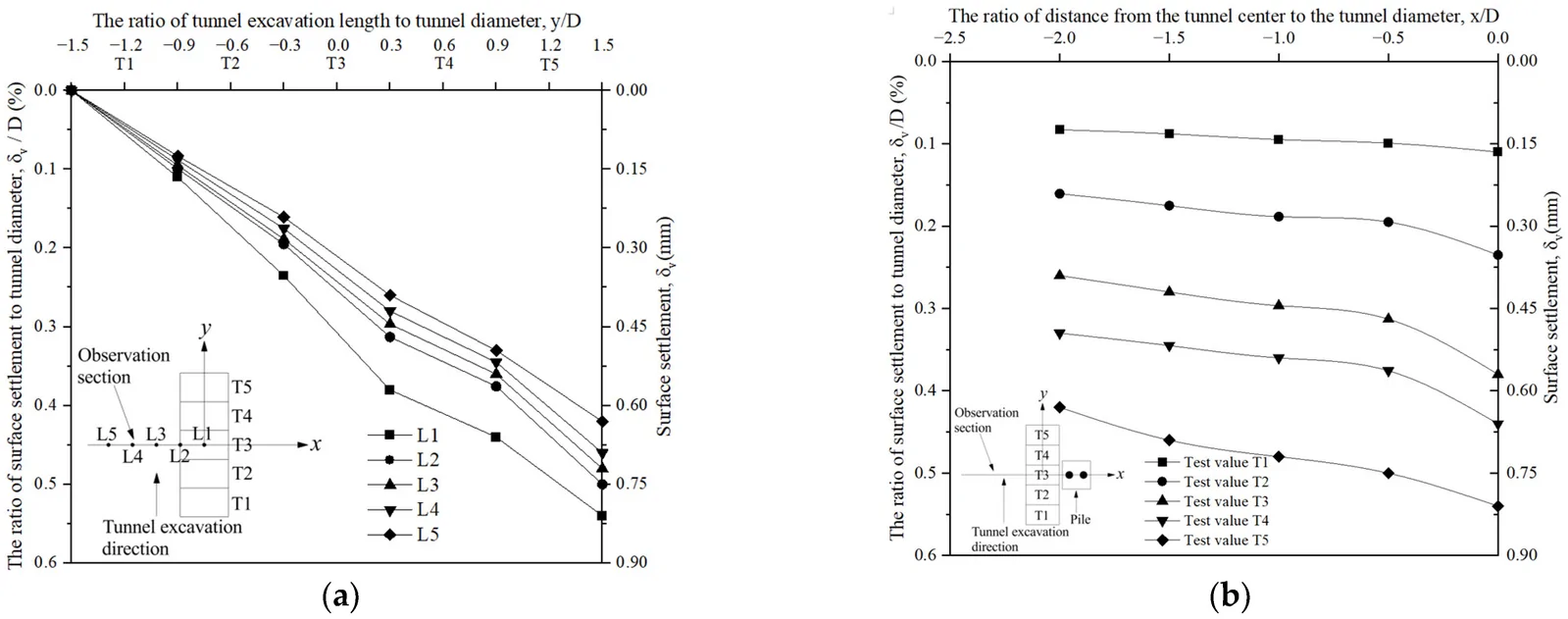
Net surface settlement caused by back tunnel excavation in test ETP: (**a**) y/D; (**b**) x/D.

As shown in Figure 10 and Figure 11 above, these represent the net surface settlement caused by tunnel excavation during the TEP and ETP experiments, respectively. The pile is located on the right side of the excavated tunnel, and displacement sensors L1-L5 are placed on the soil surface where no pile exists; this can be considered the net surface settlement caused by tunnel excavation. In the figures, x represents the horizontal distance from the tunnel axis, y represents the horizontal distance from the tunnel excavation section to the pile, and the observation section coincides with the pile centerline. The left ordinate δv/D represents the ratio of surface settlement to the tunnel diameter, and the right ordinate δv represents the actual value of surface settlement. For ease of description, this paper defines tunnel excavation into five stages based on the range of y/D: y/D = −1.5 to −0.9 is stage T1, y/D = −0.9 to −0.3 is stage T2, y/D = −0.3 to 0.3 is stage T3, y/D = 0.3 to 0.9 is stage T4, and y/D = 0.9 to 1.5 is stage T5.

As shown in Figure 10a, observation point L1 is located directly above the tunnel centerline, where the surface settlement value is the largest. In stage T1, when the tunnel is excavated towards the pile foundation, the surface settlement is relatively small, approximately 0.11%D. In stage T2, as the tunnel excavation face continues to approach the pile foundation, the surface settlement increases to 0.21%D. In stage T3, as the tunnel excavation crosses the pile foundation, the surface settlement increases significantly, reaching 0.34%D. In stage T4, the surface settlement increases to 0.43%D. Finally, in stage T5, after the tunnel excavation is completed, the surface settlement value reaches the maximum of 0.54%D (0.81 mm). As shown in Figure 10b, the transverse surface settlement troughs caused by different excavation stages exhibit the same distribution pattern. The maximum settlement occurs at the tunnel centerline, and the minimum settlement occurs at a distance of −2D from the tunnel centerline, indicating that the transverse influence range of the tunnel excavation is at least ±2D.

As shown in Figure 11a, the surface settlement under ETP condition exhibits a similar variation pattern with the tunnel excavation length as in TEP. Taking observation point L1 as an example, the surface settlement occurring in stages T2 to T4 accounts for approximately 61% of the total settlement. After the tunnel excavation is completed in stage T5, the surface settlement reaches its maximum of 0.54%D (0.81 mm), which is basically the same as the total surface settlement during tunnel excavation in TEP. However, the cumulative surface settlement during stages T2 to T4 in ETP is 3% higher than that in TEP, indicating a more concentrated increase in surface settlement. As shown in Figure 11b, the surface settlement trough under ETP condition exhibits a similar distribution pattern to that under TEP condition. The maximum settlement also occurs at the tunnel centerline, and the minimum settlement is also located at −2D from the tunnel centerline, indicating that the lateral influence range of tunnel excavation is at least ±2D. Comparing the TEP and ETP conditions, the surface settlement curves caused by tunnel excavation both conform to a typical normal distribution. The settlement curves are V-shaped, and the width of the settlement trough and the settlement value are basically the same, indicating that TEP and ETP have negligible difference in their impact on surface settlement.

#### 3.2.2. Surface Deformation Caused by Excavation

**Figure 12:**
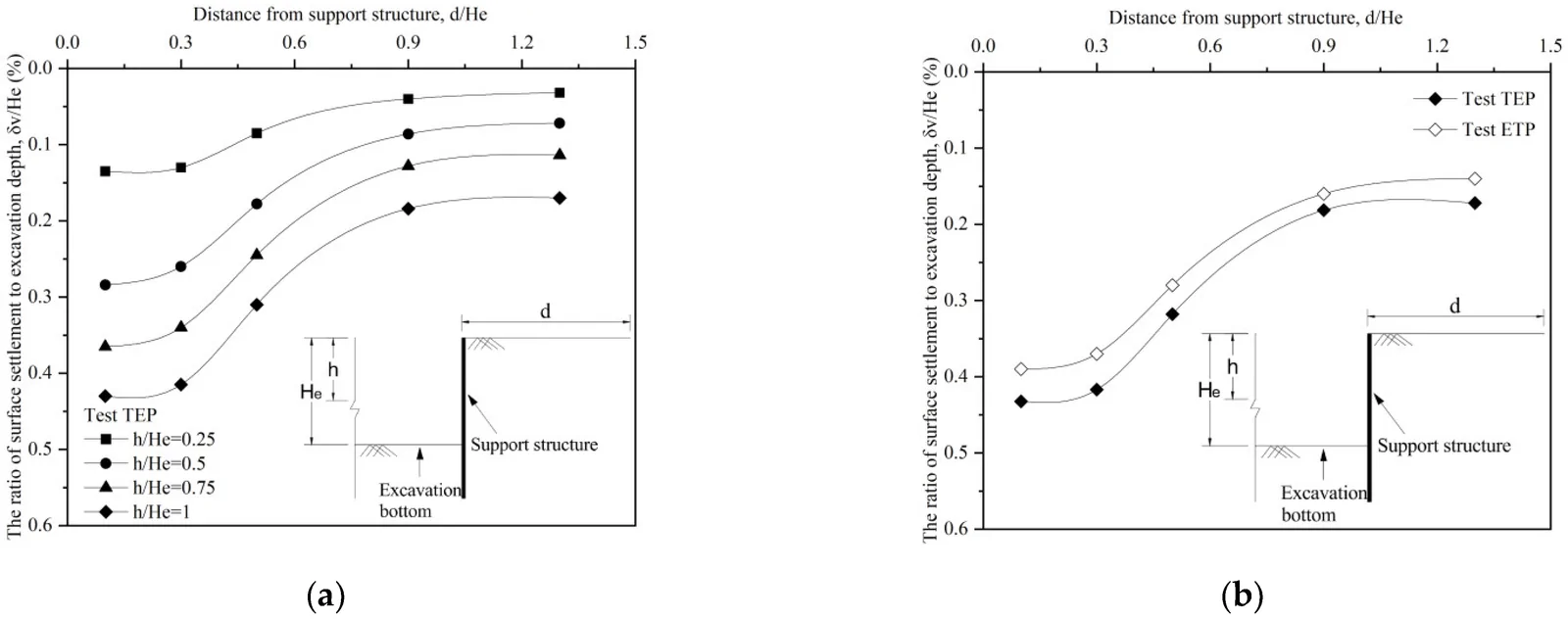
Net surface settlement caused by excavation in test TEP and ETP: (**a**) TEP; (**b**) ETP.

Figure 12a shows the net surface settlement caused by excavation in the TEP test. For ease of analysis, the surface settlement δ_v_ and the distance d from the support structure were normalized with the excavation depth He. As shown in the figure, the surface settlement around the excavation is arched, and the surface settlement is the largest near the outside of the support structure, and decreases as the distance from the support structure increases. The above settlement pattern is consistent with the typical distribution type of surface settlement around the excavation mentioned by Hsieh & Ou [24] when using no internal support. As the depth of the excavation increases, the surface settlement around the excavation also gradually increases. This is because the excavation unloading leads to the release of soil stress, and the active earth pressure behind the wall acts on the support structure to deform it, which in turn leads to the deformation of the soil behind the wall. When the excavation is completed, the maximum surface settlement is 0.43%H_e_, and the minimum surface settlement is located at 1.3 H_e_ from the support structure, with a size of 0.17%H_e_.

Figure 12b shows the comparison of net surface settlement caused by excavation in the TEP and ETP experiments. The net surface settlement in TEP is the total surface settlement after excavation minus the final cumulative settlement at the same location caused by the earlier tunnel excavation. The net surface settlement in ETP is the total surface settlement after excavation minus the final cumulative settlement at the same location caused by the earlier pile foundation loading. As can be seen from the figure, the net surface settlement caused by excavation in TEP is slightly larger than that in ETP. This may be because the excess pore water pressure generated in the soil due to the earlier tunnel excavation has not completely dissipated. As the excavation continues, the previously generated excess pore water pressure continues to dissipate over time, leading to continuous surface deformation and thus a larger net surface settlement in TEP. However, the final surface settlement of the two tests differs by only 9%, suggesting that TEP and ETP have a relatively small impact on surface settlement.

### 3.3. Pile Top Settlement

**Figure 13:**
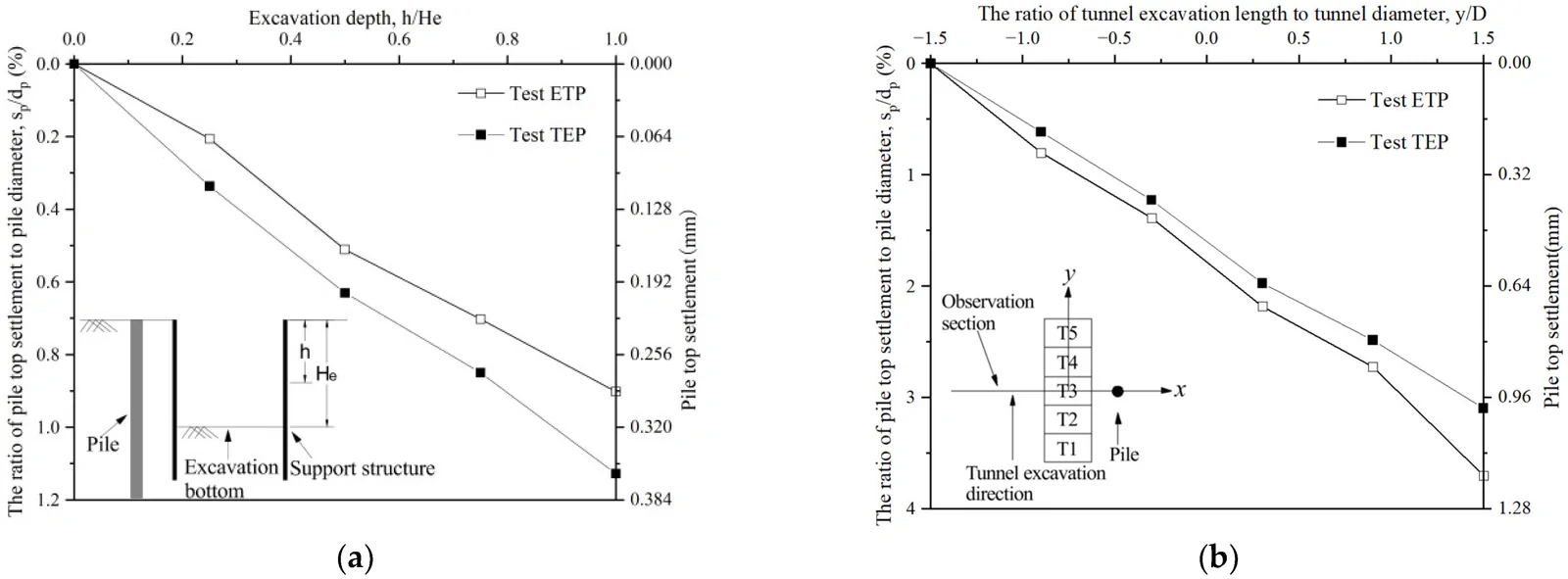
Comparison of pile top settlement in test TEP and ETP: (**a**) excavation only; (**b**) tunnel excavation only.

Figure 13 shows the net settlement at the pile top caused by excavation only in the TEP and ETP experiments in this paper. As shown in Figure 13a, the variation pattern of net pile top settlement induced by the excavation stage is basically similar between TEP and ETP. However, the net settlement at the pile top caused by excavation first in the ETP experiment is 0.90% *d_p_* (0.29 mm), which is about 20% smaller than the net settlement at the pile top caused by excavation later in the TEP experiment (1.13% *d_p_* (0.36 mm). The reasons for this may be that the tunnel excavation in the TEP test led to the transfer of pile load, and the distribution of pile load before the excavation was different in the two sets of tests; it may also be that the excess pore water pressure generated in the soil around the pile due to the tunnel excavation in the TEP test has not completely dissipated. As excavation proceeds, the excess pore water pressure continues to dissipate over time, and the soil around the pile continues to deform, resulting in a larger net settlement of the pile top caused by the subsequent excavation in the TEP test.

As shown in Figure 13b, the net pile top settlement induced by the tunnelling stage follows a similar pattern in both tests. However, the net pile top settlement induced by tunnelling as the first stage of the TEP sequence is 3.10% *d_p_* (0.99 mm), which is about 16% smaller than that induced by tunnelling as the second stage of the ETP sequence at 3.70% *d_p_* (1.18 mm). Similarly, this phenomenon may be due to the fact that the pile load was transferred because the excavation was carried out first in the ETP experiment, and the distribution of the pile load before tunnel excavation was different in the two sets of experiments; it may also be because the excess pore water pressure generated in the soil around the pile due to the excavation first in the ETP experiment has not completely dissipated. As the tunnel is excavated, the previously generated excess pore water pressure continues to dissipate over time, and the soil around the pile continues to deform, resulting in a larger net settlement at the pile top caused by tunnel excavation later in the ETP experiment. In summary, the net settlement of the pile tops after changing the excavation sequence of the tunnel and excavation in the TEP and ETP experiments is basically similar. However, due to the different load distribution characteristics of the pile foundation before excavation, the settlement of the pile foundation also shows certain differences.

**Figure 14:**
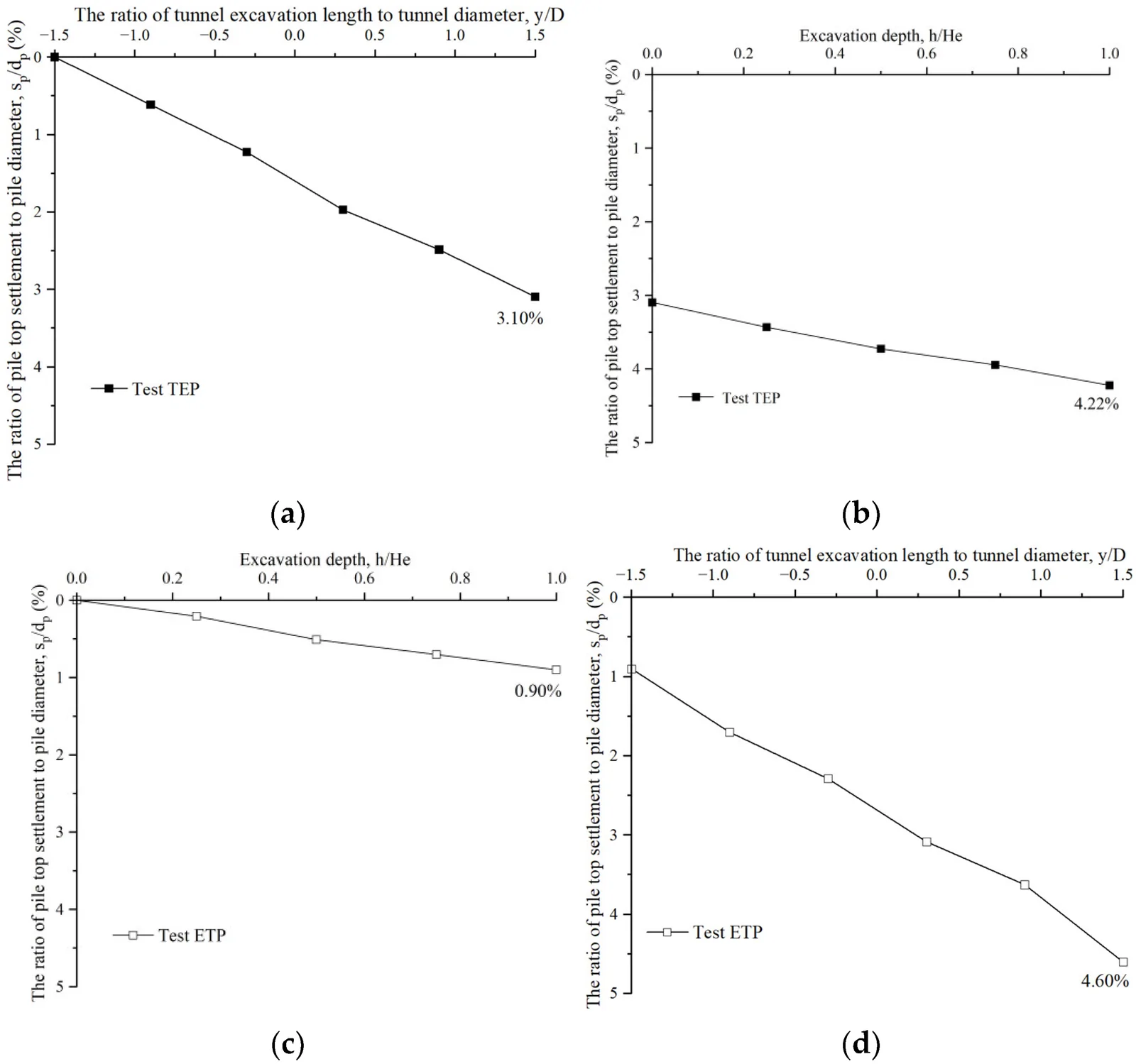
Comparison of cumulative settlement of pile top in test TEP and ETP: (**a**) y/D in test TEP; (**b**) h/He in test TEP; (**c**) h/He in test ETP; (**d**) y/D in test ETP.

Further, we can obtain the cumulative settlement of the pile top caused by the tunnel excavation in Figure 14. As shown in the figure, the cumulative settlement amounts of the pile top caused by the tunnel excavation in the two experimental groups, TEP and ETP, were 4.22% *d_p_* (1.35 mm) and 4.60% *d_p_* (1.47 mm), respectively. The corresponding settlement amounts for the prototypes were 54 mm and 58.8 mm, respectively, which, according to the classification standards for damage levels of buildings and structures, belong to the moderate damage level. In summary, the cumulative pile top settlement in the ETP test is about 9% larger than that in the TEP test, meaning that ETP leads to greater total pile top settlement. In other words, for projects where both tunnelling and excavation are conducted around existing pile foundations, especially when the spatial conditions are close to those in this study, TEP is recommended as it is more conducive to ensuring the safety of existing pile foundations.

### 3.4. Axial Force of Pile Body

**Figure 15:**
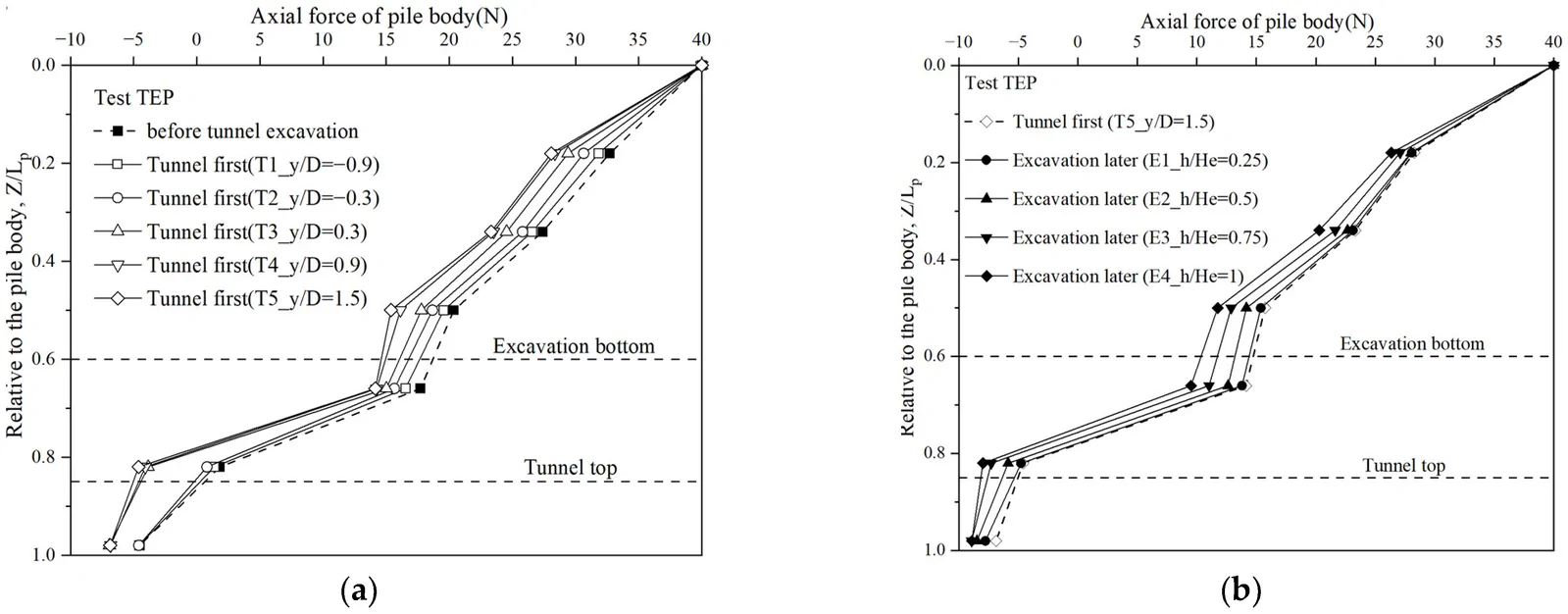
Axial force of pile shaft caused by excavation in test TEP: (**a**) tunnel first; (**b**) excavation later.

As shown in Figure 15a, before tunnel excavation, except for the negative axial force near the 0.98 *L_p_* point close to the pile tip, the axial force of the pile body showed a decreasing trend from top to bottom, indicating that the side friction resistance of the pile body was fully utilized. The reason for the negative axial force near the pile tip may be that the water level in this experiment was flush with the soil surface. The clay in this test can be considered saturated, with high sensitivity. Under the influence of pile loading, the excess pore water pressure in the soil near the pile tip increased significantly, enhancing clay thixotropy. This caused the soil near the pile tip to displace downwards relative to the pile body, generating negative skin friction. In the initial stages (T1 and T2) of tunnel excavation, near the 0.86 *L_p_* mark from the pile top to the pile shaft, pile settlement exceeds soil settlement, allowing the positive skin friction of the pile to be fully utilized, and the axial force gradually decreases. From 0.86 *L_p_* to near the pile tip, where the pile is close to the tunnel, the soil settlement near the pile tip exceeds that of the pile shaft due to tunnel excavation unloading, resulting in a slight increase in negative skin friction, which is reflected in the slightly increased negative axial force shown in the figure. In stage T3, where the tunnel passes through the pile foundation, a negative axial force is generated at 0.82 *L_p_*, indicating that the area where soil settlement near the pile tip exceeds pile settlement has further expanded. In stages T4 and T5, as the tunnel moves further away, near the 0.78 *L_p_* mark from the pile top to the pile shaft, pile settlement exceeds soil settlement, further utilizing the positive skin friction and further decreasing the axial force. From 0.78 *L_p_* to near the pile bottom, pile settlement is less than soil settlement, resulting in a slight increase in negative skin friction and a slightly increased negative axial force.

As shown in Figure 15b, with the phased excavation of excavation, the axial force gradually decreases from the pile top to near 0.78 *L_p_* of the pile shaft. From 0.78 *L_p_* to the pile tip, the negative axial force increases, indicating an increase in negative skin friction and an expansion of the negative skin friction area. In stage E1 of the excavation, the axial force changes significantly within the range of 0.18 *L_p_* to 0.82 *L_p_* of the pile shaft. As the excavation continues, the axial force further decreases, with a more significant decrease in stages E3 and E4. The reason for the decrease in axial force may be due to slippage between the pile and the soil, resulting in pile settlement exceeding soil settlement. This allows the pile’s side skin friction to be fully utilized, leading to a decrease in axial force and upward load transfer.

The maximum axial force was established through the single-pile bearing capacity test, as described in Section 3.1. Based on the results presented in Figure 9, the peak axial force for the single pile is 60 N.

**Figure 16:**
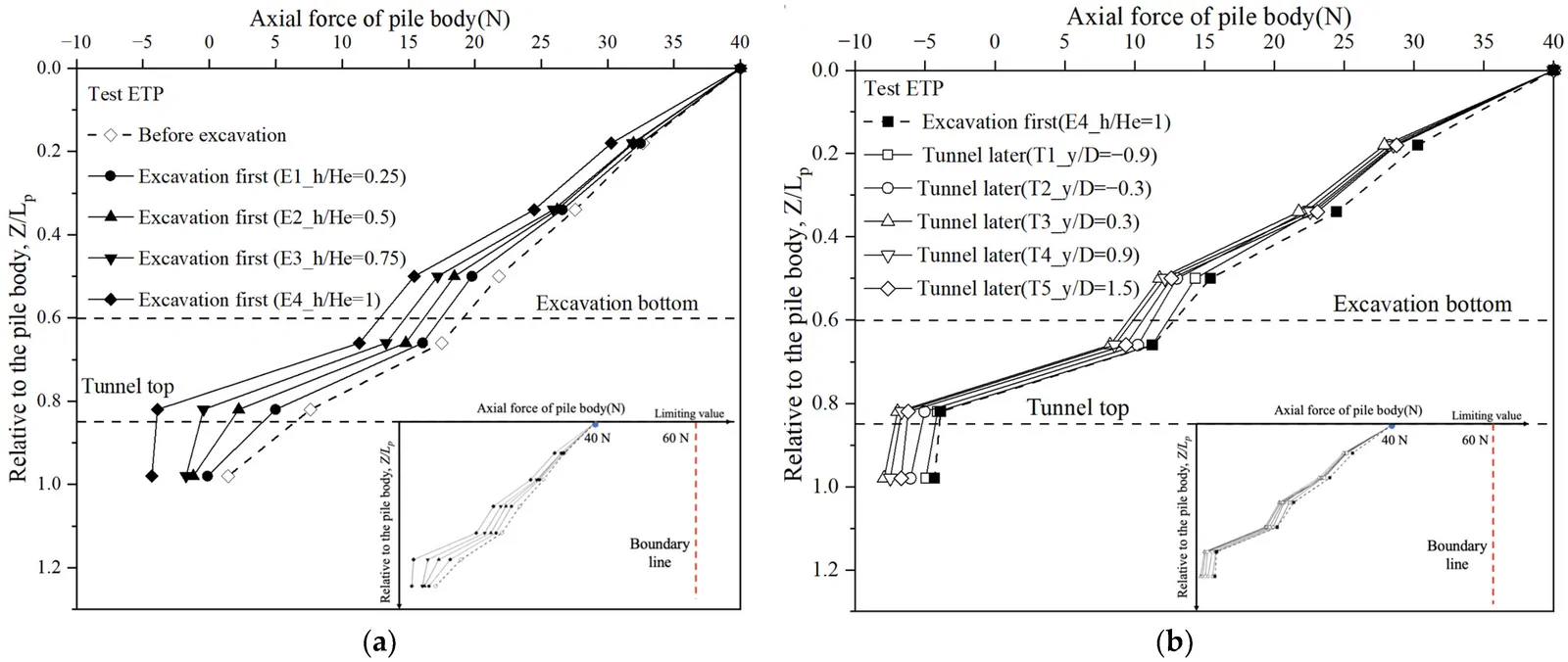
Axial force of pile shaft caused by excavation in test ETP: (**a**) excavation first; (**b**) tunnel later.

Figure 16a shows the change in pile axial force caused by excavation first in the ETP experiment, which is basically consistent with the change in axial force caused by the subsequent excavation in the TEP experiment. As the excavation proceeds layer by layer, the pile axial force shows a “single decrease” state, with a larger change in axial force near the bottom of the excavation to the top of the tunnel. The reason for the decrease in axial force may also be that there is a working load on the pile top, and the excavation causes slippage between the pile and the soil, which allows the positive side friction of the pile to be fully utilized, thus reducing the axial force. Figure 16b shows the change in pile axial force caused by the subsequent tunnel excavation in the ETP experiment. Unlike the “single decrease” pattern of axial force caused by tunnel excavation first in the TEP experiment, the axial force shows a decreasing trend in stages T1 to T3, while it shows an increasing trend in stages T4 and T5. The reason for this may be due to the different load distribution patterns of the piles before tunnel excavation. The excavation has a certain influence on the axial force variation caused by subsequent tunnel excavation. Specifically, the tunnel excavation in stages T4 and T5 may result in a more significant release of stress in the surrounding soil, leading to a decrease in the normal stress acting on the piles, a decrease in side friction, and a downward transmission of the load, resulting in an increase in the axial force of the piles.

In summary, the construction sequence has a notable impact on the variation in pile axial force. For TEP, both tunnelling and excavation stages lead to a decrease in pile axial force. For ETP, the axial force reduction pattern during the excavation stage remains similar; however, during the subsequent tunnelling stage, the axial force decreases in stages T1–T3 and increases in stages T4–T5.

### 3.5. Pile Bending Moment

**Figure 17:**
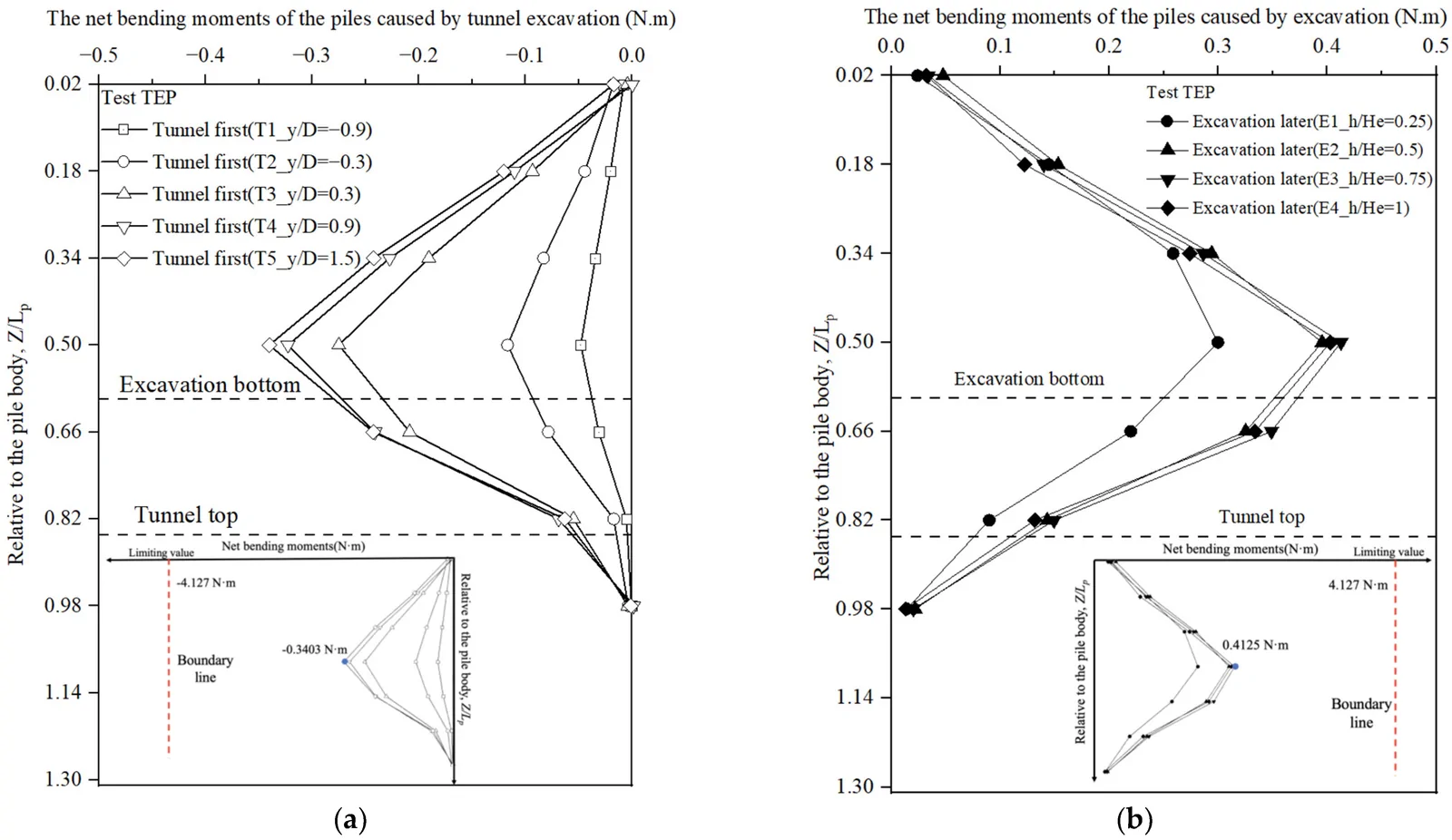
Net bending moment of pile shaft caused by test TEP: (**a**) the net bending moments of the piles caused by tunnel excavation first; (**b**) the net bending moments of the piles caused by excavation later.

Figure 17 shows the net bending moments of the piles caused by the tunnel excavation followed by the excavation in the TEP experiment presented in this paper. The horizontal axis represents the net bending moments of the piles caused by the tunnel excavation and the subsequent excavation, respectively, and the vertical axis represents the relative pile *Z/L_p_*. The net bending moment caused by the subsequent excavation is not actually present; it cancels out the bending moment caused by the tunnel excavation. In this paper, tension on the pile surface near the tunnel is defined as a positive bending moment, and compression is defined as a negative bending moment.

The yield bending moment was determined using elastic beam bending theory $M_{y}=\frac{F_{y}I_{z}}{y_{\max}}$, outer diameter *d_p_* = 32 mm, inner diameter *d_i_* = 28 mm; $I_{z}=\;\frac{\pi (d_{p}^{4}-d_{i}^{4})}{64}$, evaluated for a hollow circular cross-section; $y_{\max}=\;d_{p}/2$, $F_{y}=\;3100\;\mathrm{MPa}$, matching the mechanical parameter used for PMMA retaining components in Section 2.2.3. By substituting all geometric and material parameters into the flexure formula, the elastic limit bending moment of the model pile was determined as 4.127 N·m.

As shown in Figure 17a, during the five-step tunnel excavation process, the pile body generated a negative bending moment, with the maximum value of −0.34 N·m occurring near the 0.50 *L_p_* point of the pile. This is likely due to the unloading effect of tunnel excavation, which relaxes the soil stress near the pile, causing horizontal compression of the pile foundation by the soil on the right side, resulting in a negative bending moment on the pile surface near the tunnel. The pile tip to the top can be approximated as a simply supported connecting beam, with the bending moment at both ends being essentially zero, reaching its maximum value near the midpoint of the pile. From the perspective of the tunnel excavation stages, the T3 stage, where the tunnel crosses the pile foundation, exhibits the largest change in net bending moment, indicating the danger of tunneling through the pile foundation and the need for special attention during construction. While the bending moment continues to increase in stages T4 and T5 compared to stages T1 and T2, the change in net bending moment is smaller compared to stages T1 and T2, where the tunnel excavation face is close to the pile foundation.

As shown in Figure 17b, the net bending moment of the pile body generated during the four-step excavation of the excavation shows an opposite trend to that generated by the tunnel excavation. The pile body as a whole generates a positive bending moment, with the maximum value of 0.41 N·m near 0.50 *L_p_* of the pile body. The reason for this is that the excavation and unloading of the excavation causes slight deformation of the support structure into the excavation, resulting in relaxation of soil stress near the pile body. The soil on the left side of the pile horizontally compresses the pile foundation, causing tension on the pile surface near the tunnel side, generating a positive bending moment. Since there is no constraint at the pile top and bottom, the resulting bending moment is essentially close to zero. From the perspective of the excavation stages, the E1 stage (excavation of the first layer of soil) causes the largest change in the net bending moment of the pile body. The E2 stage (excavation of the second layer of soil) shows the second largest change. Although the net bending moment of the pile body continues to increase in stages E3 and E4, the changes are small and tend to stabilize.

**Figure 18:**
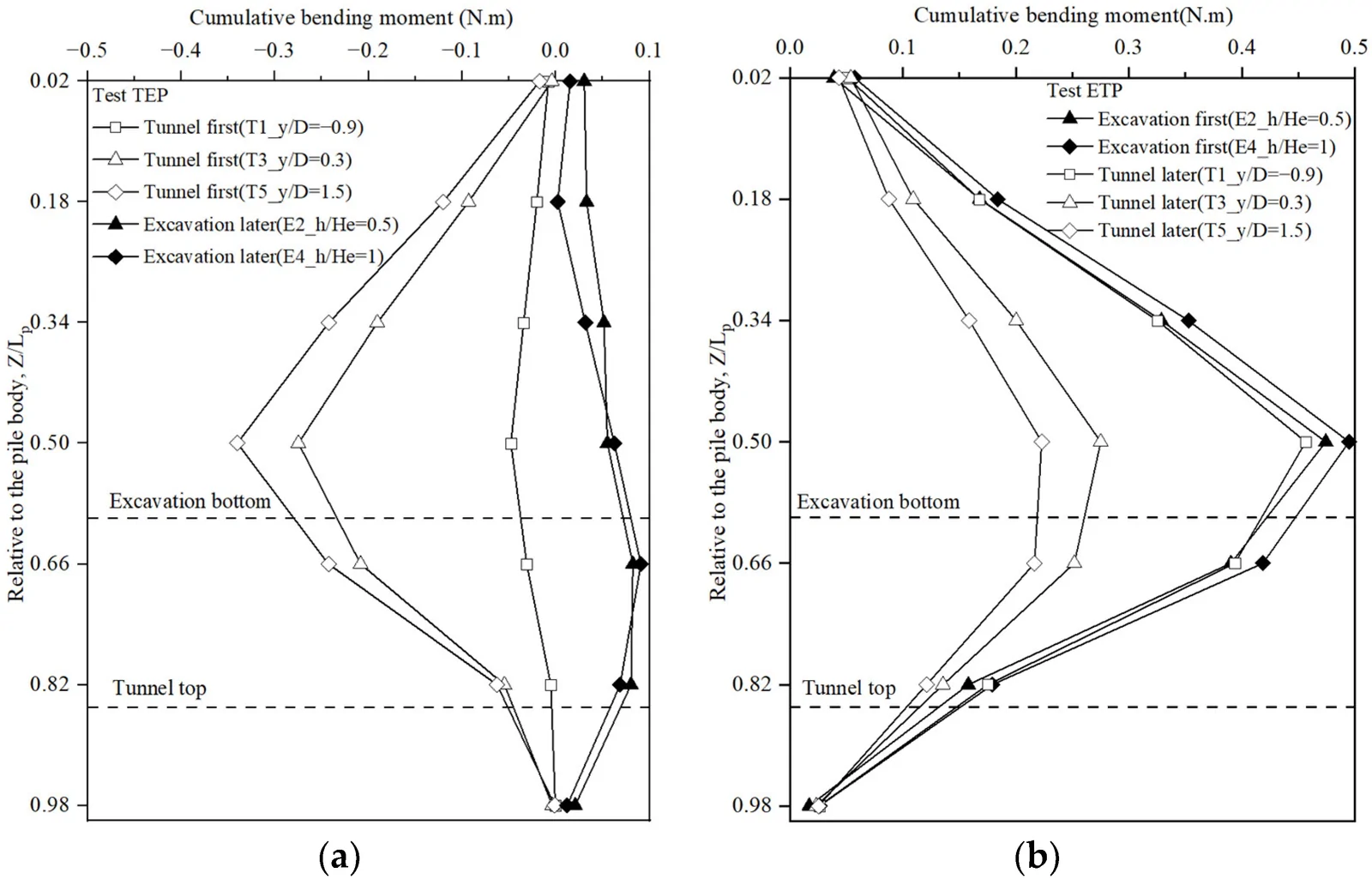
Comparison of pile cumulative bending moment in test TEP and ETP: (**a**) TEP; (**b**) ETP.

Figure 18 shows the cumulative bending moment of the piles at each stage of the TEP and ETP experiments. As shown in Figure 18a, the cumulative bending moment was largest at the end of the TEP tunnel excavation, reaching 0.34 N·m. With subsequent excavation, the cumulative bending moment turned positive because the excavation caused more severe stress relaxation in the soil around the piles, resulting in horizontal compression of the pile foundation by the soil on the left side. This caused tension and a larger positive bending moment on the side of the pile foundation closer to the tunnel, offsetting the negative bending moment caused by the earlier tunnel excavation. As shown in Figure 18b, the cumulative bending moment was largest at the end of the ETP excavation, reaching 0.49 N·m. With segmented tunnel excavation, the bending moment gradually decreased because the excavation caused more severe stress relaxation in the soil around the piles, while the pile foundation remained under horizontal compression from the soil on the left side and under tension on the side closer to the tunnel, resulting in a consistently positive bending moment.

In summary, for the TEP and ETP tests, the distribution patterns of net bending moments of the piles caused by tunnel excavation alone and excavation alone are basically similar. The maximum cumulative bending moment in the TEP test is 31% smaller than that in the ETP test, which further indicates that TEP is more beneficial to the safety of existing pile foundations. However, in general, the bending moment of the piles caused by unloading during tunnel excavation is smaller in the tests.

### 3.6. Excess Pore Water Pressure

**Figure 19:**
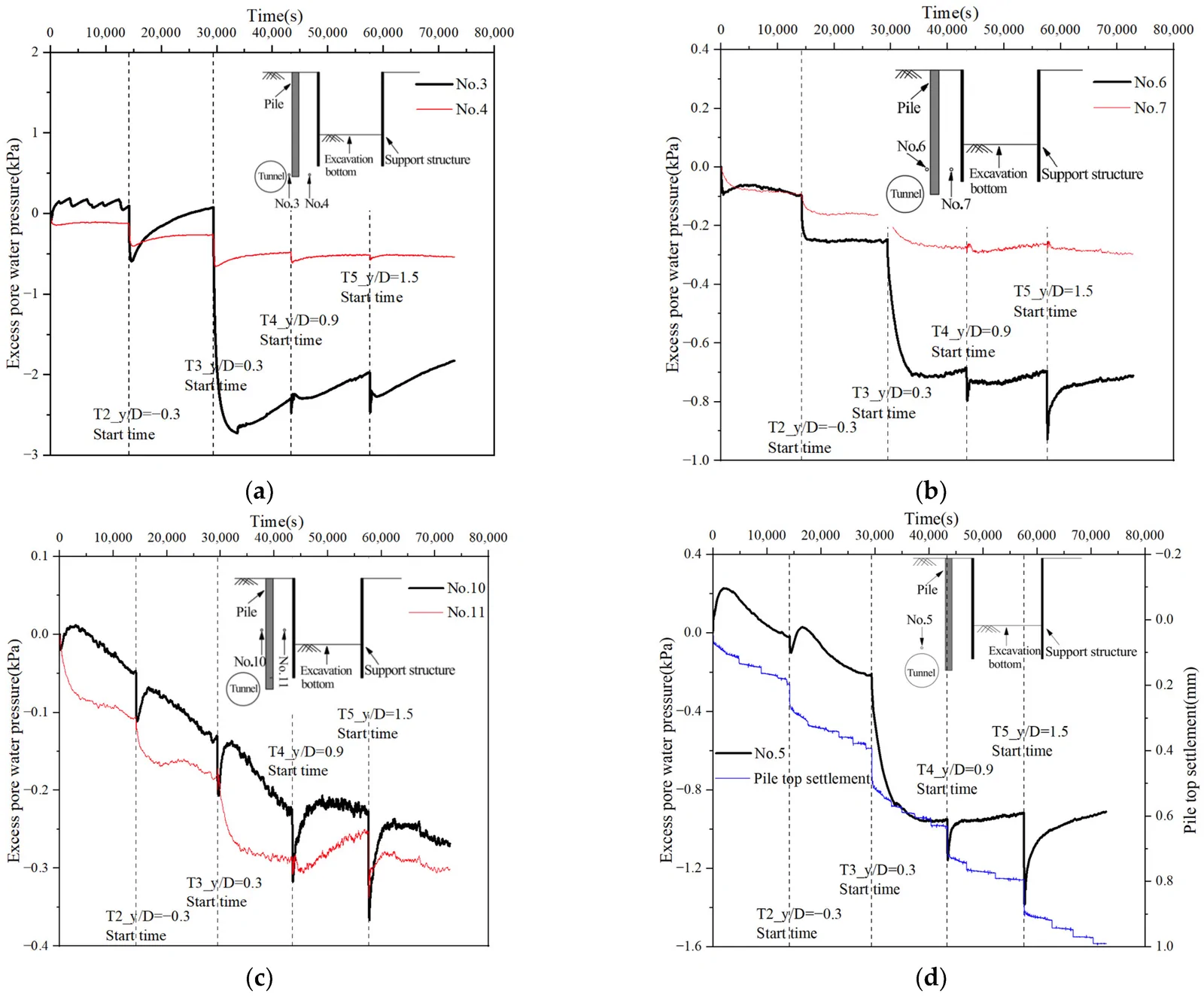
Variation in pore water pressure caused by tunnel excavation in test TEP: (**a**) pore pressure variations in Boreholes 3 and 4; (**b**) pore pressure variations in Boreholes 6 and 7; (**c**) pore pressure variations in Boreholes 10 and 11; (**d**) pore pressure variations in Borehole 5.

Figure 19 shows the changes in pore pressure around the existing pile foundation caused by tunnel excavation in the TEP experiment of this paper. The horizontal axis represents time, and the vertical axis represents excess pore water pressure. By comparing and observing the changes in pore pressure over time, the law of pile foundation response as excess pore water pressure dissipates over time was obtained. It should be noted that the next stage of tunnel excavation did not wait for the excess pore water pressure caused by the previous stage to completely dissipate before proceeding to the next stage. Instead, the next stage of tunnel excavation was carried out after the pile settlement and surface settlement had basically stabilized.

As shown in Figure 19a, the tunnel excavation as a whole will cause negative excess pore water pressure in the soil, and the negative excess pore water pressure will dissipate over time, with the dissipation rate becoming increasingly slower. Pore gauge No. 3 is located on the left side of the pile end. In the T1 stage of tunnel excavation, a positive excess pore water pressure of approximately 0.20 kPa is generated. This may be due to the volume loss of soil caused by the excavation of the first section of the tunnel. There is a certain cohesion between the clay, and the pore water in the nearby soil preferentially flows into the volume loss area of the tunnel. However, at this time, the pore water flowing in has not yet affected the No. 3 pore gauge at the center of the third section of the tunnel. At the same time, with the subsidence of the soil above the tunnel and the movement of the soil on the side towards the tunnel, the water flowing into the volume loss area of the tunnel is squeezed. The water slowly seeps into the surrounding soil, causing the pore water pressure in the nearby soil to increase, generating positive excess pore water pressure. During stage T3, when the tunnel passes through the pile foundation, the changes in the values of pore pressure gauges No. 3 and No. 4 are most significant. This is because the excavation face is closest to the pore pressure gauges at this stage. The excavation of the third tunnel section causes soil volume loss, and pore water in the nearby soil flows into the area of tunnel volume loss, resulting in a significant decrease in pore pressure within the soil. Although both pore pressure gauges No. 3 and No. 4 are located at the pile tip, the negative excess pore water pressure of pore pressure gauge No. 3 is significantly greater than that of No. 4. This is because No. 3 is closer to the tunnel, and due to the presence of the existing pile foundation, the seepage path of water in the soil on the right side of the pile is longer, resulting in slower pore water loss from the soil.

In Figure 19b, the excess pore water pressure generated by gauge 6 is negative. With each excavation of the tunnel, the negative excess pore water pressure reaches its maximum value within a short period, and then slowly dissipates over time. The change in excess pore water pressure is most significant in stage T3. Furthermore, the negative excess pore water pressure value of gauge 6 on the left side of the pile is greater than that of gauge 7 on the right side, likely because gauge 6 is closer to the tunnel and has no existing pile foundation obstruction. The figure shows that the negative excess pore water pressure of gauge 7 dissipates extremely slowly, possibly due to the influence of the excavation support structure embedded in the soil.

As shown in Figure 19c, the pore pressure of gauge 11 is basically decreasing from stage T1 to T3. This is because the presence of the existing pile foundation and the pre-embedded excavation support structure causes the excess pore water pressure generated in stages T1 to T3 to dissipate extremely slowly. Gauge 10, located on the left side of the pile, shows a trend of “increasing first, then decreasing, then slowly increasing again” after each excavation of the tunnel.

In Figure 19d, pore pressure gauge No.5 directly above the third excavation section of the tunnel begins to generate positive excess pore water pressure during stages T1 and T2, similar in principle to the positive excess pore water pressure generated by pore pressure gauge No.3 on the left side of the pile tip. With each tunnel excavation, the negative excess pore pressure and pile top settlement increase significantly; the pore pressure dissipates over time while the pile top settles.

Comparing the excess pore water pressures of gauges 3, 6, and 10, or 5, 6, and 7, it is evident that the closer to the tunnel, the greater the impact and the greater the negative excess pore water pressure generated. Before monitoring ceased, the excess pore water pressure generated by tunnel excavation had not completely dissipated, but the pile settlement and surface settlement remained relatively stable. As the remaining excess pore water pressure dissipates, it still has a certain impact on the pile foundation and the ground surface. Therefore, long-term monitoring of adjacent existing structures is critical for tunnelling in clay.

**Figure 20:**
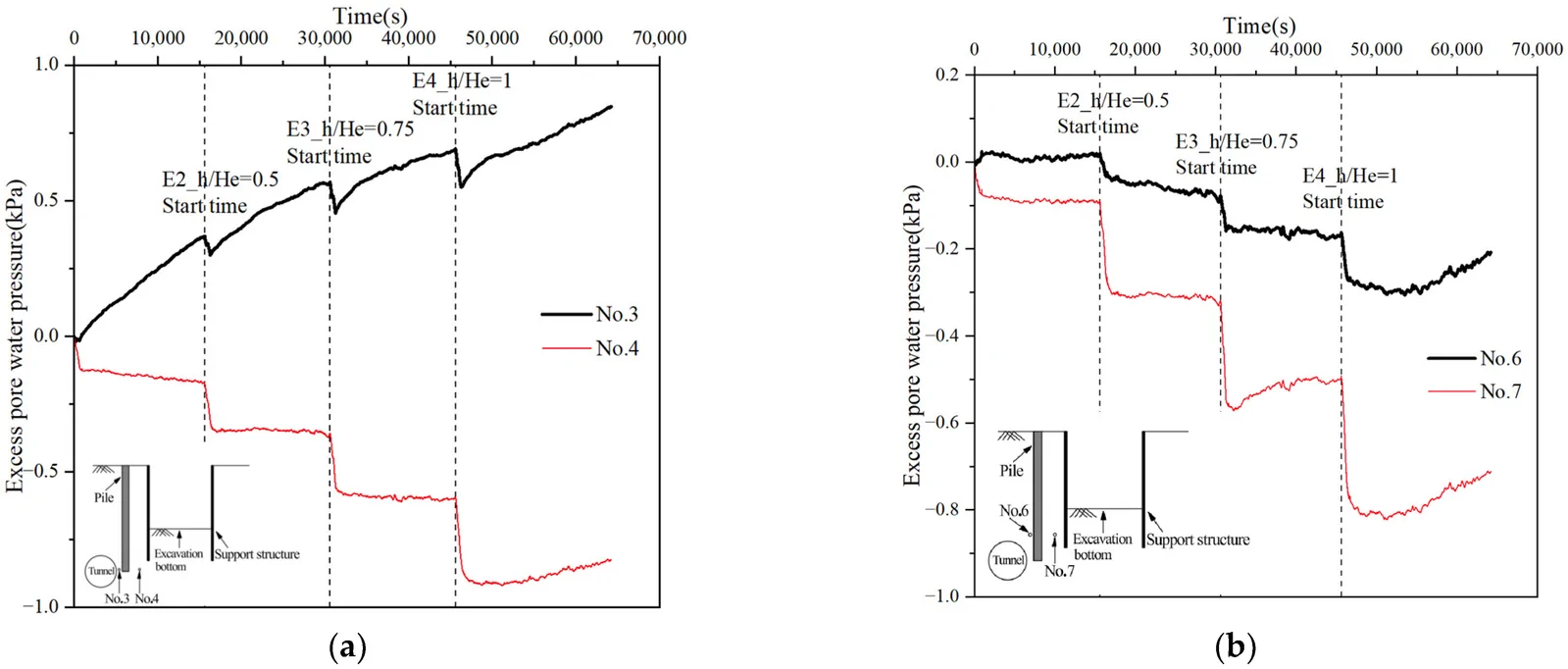

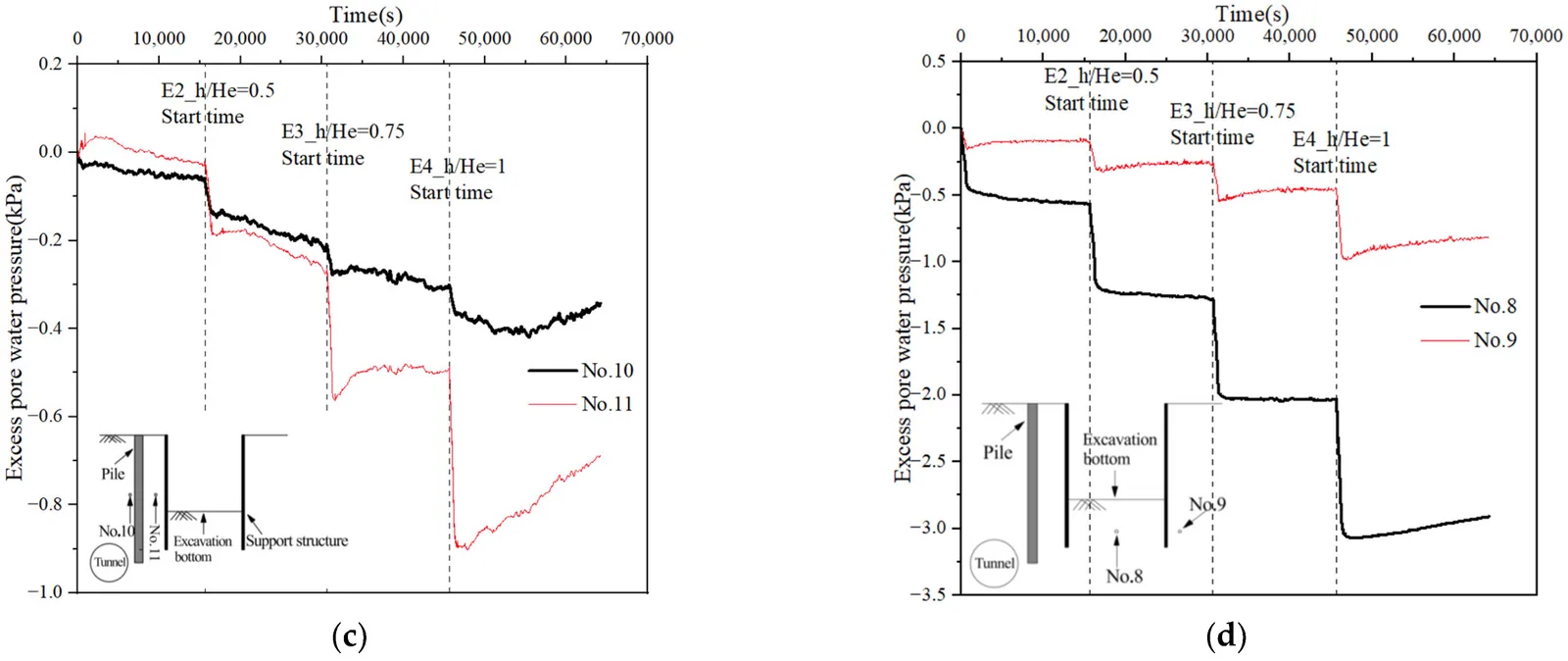
Variation in pore water pressure caused by excavation in test TEP: (**a**) pore pressure variations in Boreholes 3 and 4; (**b**) pore pressure variations in Boreholes 6 and 7; (**c**) pore pressure variations in Boreholes 10 and 11; (**d**) pore pressure variations in Boreholes 8 and 9.

Figure 20 shows the changes in pore pressure around the existing pile foundation and around the excavation caused by excavation during the TEP experiment. The horizontal axis represents time, and the vertical axis represents excess pore water pressure. It should be noted that before the excavation was excavated, the excess pore water pressure caused by the tunnel excavation had not completely dissipated, which may have had a certain impact on the excess pore water pressure caused by excavation.

As shown in Figure 20a, the changes in pore pressure on the left and right sides of the pile bottom are significantly different. Pore gauge No. 3, located on the left side of the pile bottom, shows a gradual increase in excess pore water pressure as the four layers of soil in the excavation are excavated. The decrease is due to the unloading effect of the excavation, which reduces the overburden pressure at the bottom of the pit. However, the soil on the left side of the pile tip shifts to the right, causing the excess pore water pressure within the soil to increase to a positive value. The excess pore water pressure generated by pore gauge No. 4 in stages E1–E3 is extremely slow. Only after the negative excess pore water pressure further increases in stage E4 does a more significant dissipation of excess pore water pressure occur, possibly due to the increased hydraulic gradient.

Compared to pore gauge No.6, pore gauge No.7 is closer to the excavation and has no existing pile foundation obstruction, resulting in a significantly greater negative excess pore water pressure than No. 6, as shown in Figure 20b. In stage E1, pore pressure gauge No. 6 generates excess pore water pressure slightly greater than zero, similar in principle to the positive excess pore pressure generated by pore pressure gauge No.3. It can be seen that the impact of excavation on excess pore water pressure is least in stage E1 and most significant in stage E4.

As shown in Figure 20c, pore pressure gauge No. 11, due to its proximity to the excavation and the absence of existing pile foundations, generates a negative excess pore water pressure value greater than that of gauge No.10. As shown in Figure 20d, pore pressure gauge No. 8, located at the bottom of the excavation, generates the largest negative excess pore water pressure, and no significant pore pressure dissipation is observed in stages E1 to E3. This may be due to the influence of the excavation support structure, which lengthens the seepage path of water within the soil, and also because the clay has a low permeability coefficient, resulting in no significant pore pressure dissipation process in a short period. In stage E4, the negative excess pore water pressure further increases, the hydraulic gradient further increases, and the excess pore pressure can be seen slowly dissipating. Pore pressure gauge No.9, located outside the excavation support structure, shows that the negative excess pore water pressure reaches its maximum value within a short period and then slowly dissipates over time. Therefore, for tunnelling and excavation in clay, it is crucial to monitor the effects of excess pore water pressure on existing structures and ground settlement.

In summary, the closer to the tunnel excavation face or the excavation, the greater the impact of excavation and the higher the resulting negative excess pore water pressure. During tunnel and excavation construction in clay layers, because excess pore water pressure dissipates slowly and is accompanied by soil deformation, it is essential to monitor surrounding existing buildings and structures and ground settlement, and take appropriate measures to mitigate potential hazards.

## 4. Numerical Simulation Result Analysis

### 4.1. Establishment of the Three-Dimensional Model and Mesh Generation

**Figure 21:**
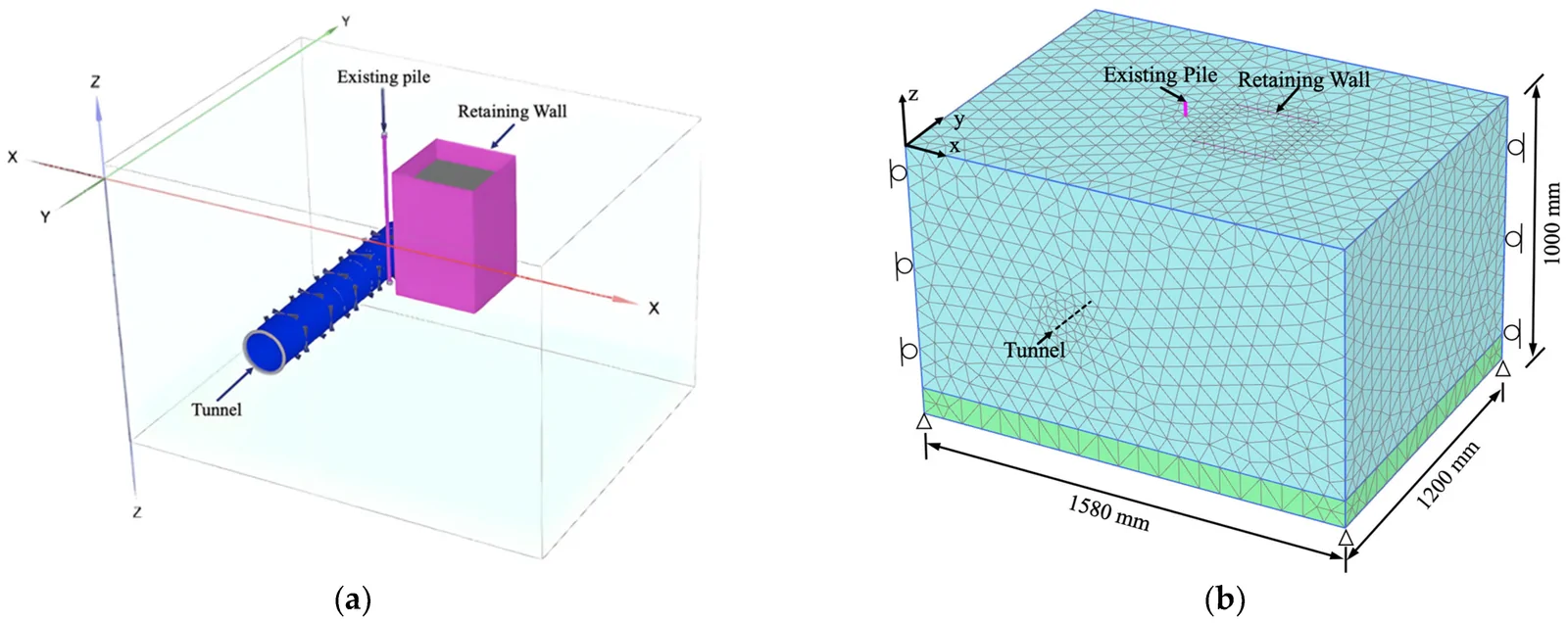
Test TEP modeling diagram: (**a**) 3D model; (**b**) model mesh generation schematic.

The 3D model is shown in Figure 21a. The mesh generation schematic based on the indoor scaled model test is presented in Figure 21b. The numerical model dimensions were identical to those of the indoor scaled model test, measuring 1580 mm in length, 1200 mm in width, and 1000 mm in height. To improve calculation accuracy, the mesh size was set to “fine”; automatic refinement was applied to the mesh around the tunnel and excavation.

Saturated clay strata are represented by three-dimensional solid elements, while tunnel linings and foundation pit retaining structures are modeled with shell elements, and the existing single pile is simulated using solid pile elements. Goodman interface elements are incorporated at the soil–structure interfaces between the pile, lining, retaining wall, and surrounding clay to capture interfacial sliding and frictional behavior. The global mesh size for the far-field soil is set to 800 mm. Local mesh refinement, with an element size of 200 mm, is applied within one tunnel diameter around the tunnel, 1.2 excavation depths beyond the pit, and 0.8 pile diameters on either side of the pile shaft, in order to accurately resolve the high-gradient stress and deformation fields in the vicinity of underground structures. The entire model comprises 126,847 solid elements, 1436 structural elements, and 921 interface elements. For displacement boundaries, the bottom surface of the model is fully fixed in the X, Y, and Z directions, the four vertical lateral boundaries only constrain their respective normal displacements, and the ground surface remains unconstrained as a free boundary. The seepage boundary conditions mirror those of the indoor model test, with the groundwater table maintained at the ground surface level.

Regarding mesh convergence, the critical zones encompassing the tunnel, excavation, and neighboring pile are discretized with substantially smaller elements than the far-field soil, thereby guaranteeing adequate numerical resolution in the region of interest. Furthermore, the simulated deformation, internal force, and excess pore water pressure results have been thoroughly validated against measured data from the indoor scaled model test in Section 3. The favorable correlation between numerical predictions and experimental observations adequately confirms that the adopted mesh scheme satisfies the accuracy requirements, serving as a dependable alternative to standalone mesh convergence studies.

### 4.2. Model and Soil Parameters

(1) Model parameters

The pile foundation was modeled with beam elements rather than embedded piles, assigning an elastic material (unit weight: 13.50 kN/m^3^) and a predefined circular tube cross-section (diameter: 0.032 m; thickness: 0.002 m; stiffness: E = 3 GPa).

The excavation support structure was modeled with plate elements. An elastic, isotropic material was defined with a unit weight of 11.80 kN/m^3^ and stiffness parameters: E1 = 3.0 GPa, v12 = 0.38, and d = 0.004 m.

Similarly, the aluminum alloy lining was modeled with plate elements. An elastic, isotropic material was assigned with a unit weight of 26.5 kN/m^3^ and stiffness parameters: E1 = 72 GPa, v12 = 0.33, and d = 0.003 m.

(2) Soil parameters

In this test, the soil height was 1.0 m. Sand occupied the lower 0.1 m, and clay occupied the upper 0.9 m. The sand layer was at the bottom, and the shallowest burial depth of the model was 0.4 m (at the top surface of the sand layer). The water level was kept constant at 0 m during the test, and the influence of the sand parameters on the test results was negligible; the parameters were taken from empirical values. The clay was taken from Hangzhou, Zhejiang Province. Referring to the relevant literature [52], the soil parameters of the HSS model for the main soil layers in Hangzhou (including fill soil, silty clay, muddy silty clay, sandy silt, clay, silty clay containing sand, etc.) were obtained. Combined with empirical parameters and the actual conditions of the test, the following parameters were obtained (See Table 2).

### 4.3. Numerical Simulation Process

(1) A basic finite element model was established (Figure 21b) with boundary and initial stress conditions defined.
(2) Consistent with the experimental procedure, the plate elements for the tunnel lining and excavation support, along with the beam elements for the pile foundation, were activated, followed by the application of a predetermined working load to the pile top.
(3) Tunnel volume loss due to excavation was simulated using the surface shrinkage method. This parameter was activated sequentially for each of the five tunnel sections until the excavation of all sections was completed.
(4) Upon completion of tunnel excavation, the soil elements in the first layer of the excavation were deactivated, and the hydraulic conditions were set to “dry” to simulate the removal of this layer. This procedure was repeated until all four soil layers were removed.
(5) Following the completion of the 3D numerical analysis, the results were extracted, processed, and analyzed.

It is worth noting that the numerical simulations accounted for the phased construction sequence. Tunnel excavation was modeled in five successive stages, while basement excavation was modeled in four successive stages, aligning with the experimental protocol. Between consecutive stages, a consolidation analysis was conducted over a specified time interval to permit dissipation of excess pore water pressure, a factor of particular significance in clay strata. The time intervals employed in the numerical model were determined based on the stabilization periods observed in the model tests.

### 4.4. Validation of a Three-Dimensional Numerical Model of the Response of a Near Pile Under Unloading During Tunnel Excavation

#### 4.4.1. Surface Settlement

**Figure 22:**
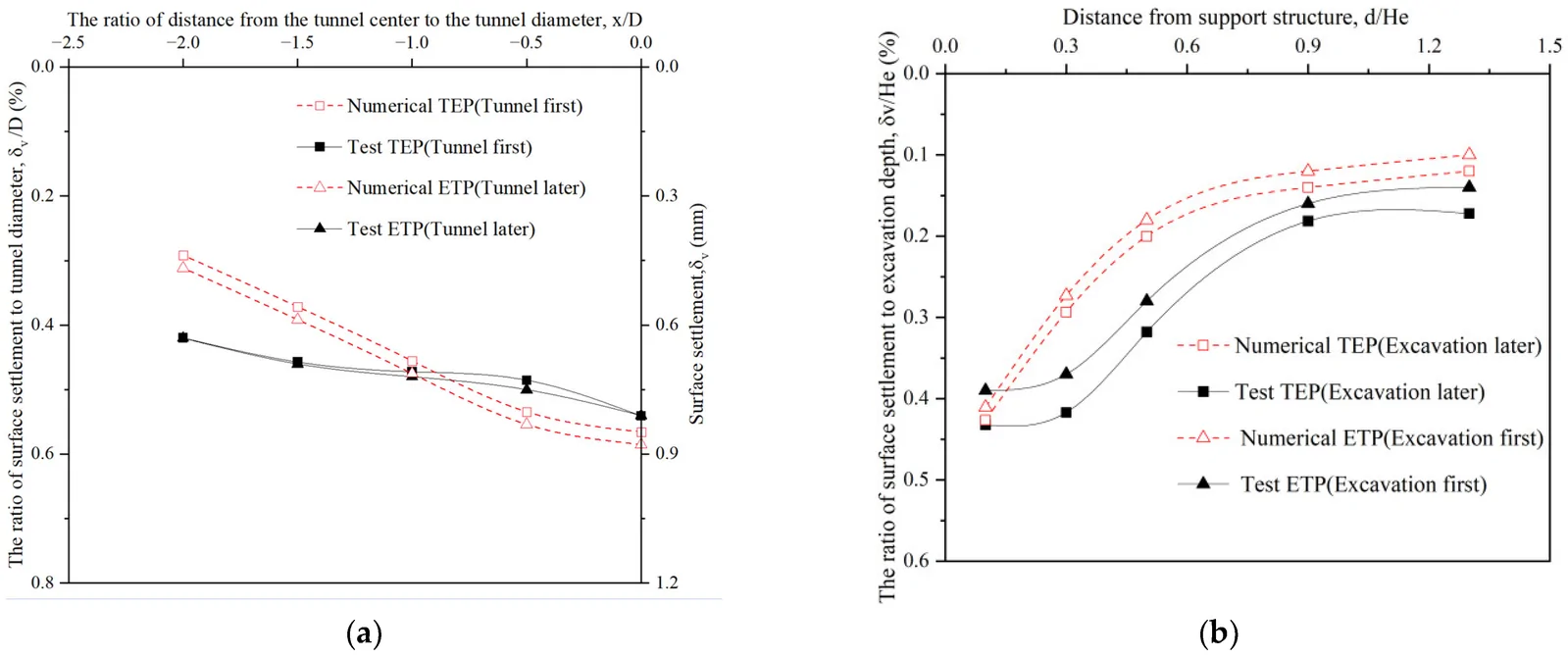
Comparison between numerical and experimental results of surface settlement: (**a**) Tunnel only. (**b**) Excavation only.

Figure 22 presents a comparison between the numerical simulation and experimental results of surface settlement for the TEP and ETP cases. As illustrated in Figure 22a, when considering solely the tunnel excavation phase, the maximum surface settlement was found to be generally consistent with the model test results. However, the settlement trough exhibited a more typical Gaussian distribution, with a width smaller than that observed in the tests. Specifically, surface settlement within a distance of 1D from the tunnel center was overestimated, while that beyond 1D was underestimated, which may be attributed to soil anisotropy. Figure 22b indicates that the numerical results follow a similar trend to the model tests. The maximum settlement was observed near the excavation support structure, decreasing with increasing distance. Overall, the numerical values were slightly lower than the experimental measurements.

**Figure 23:**
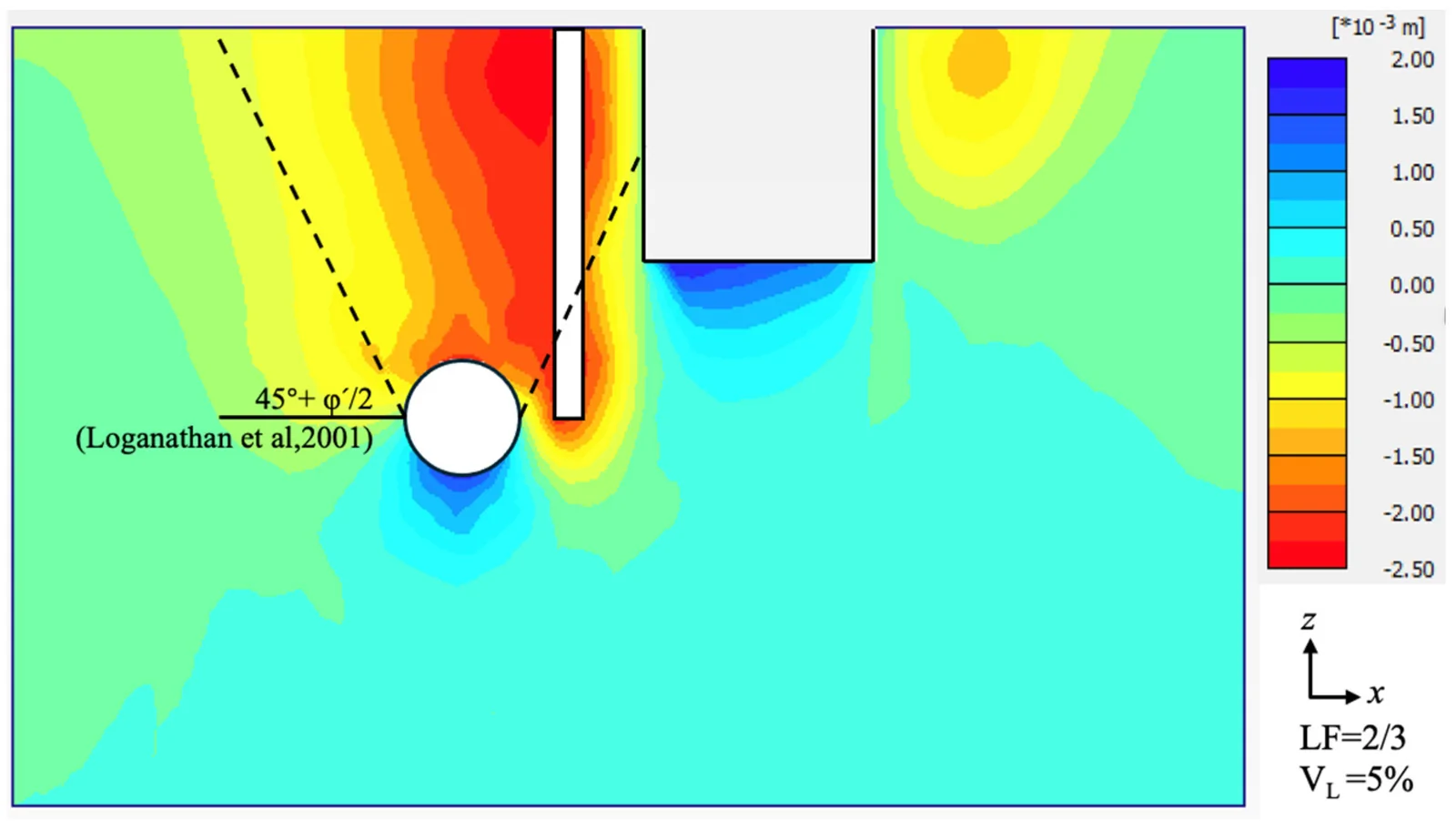
Vertical displacement contour plots of the soil for test ETP [53].

Figure 23 presents the vertical displacement contour map for the ETP case. The primary influence zone of the surrounding soil due to tunnel excavation aligns well with the theoretical predictions of Loganathan et al. [53]. This zone originates from the tunnel arching line and propagates upward at an angle of 45 + Φ’/2 relative to the horizontal plane until reaching the ground surface.

#### 4.4.2. Pile Top Settlement

**Figure 24:**
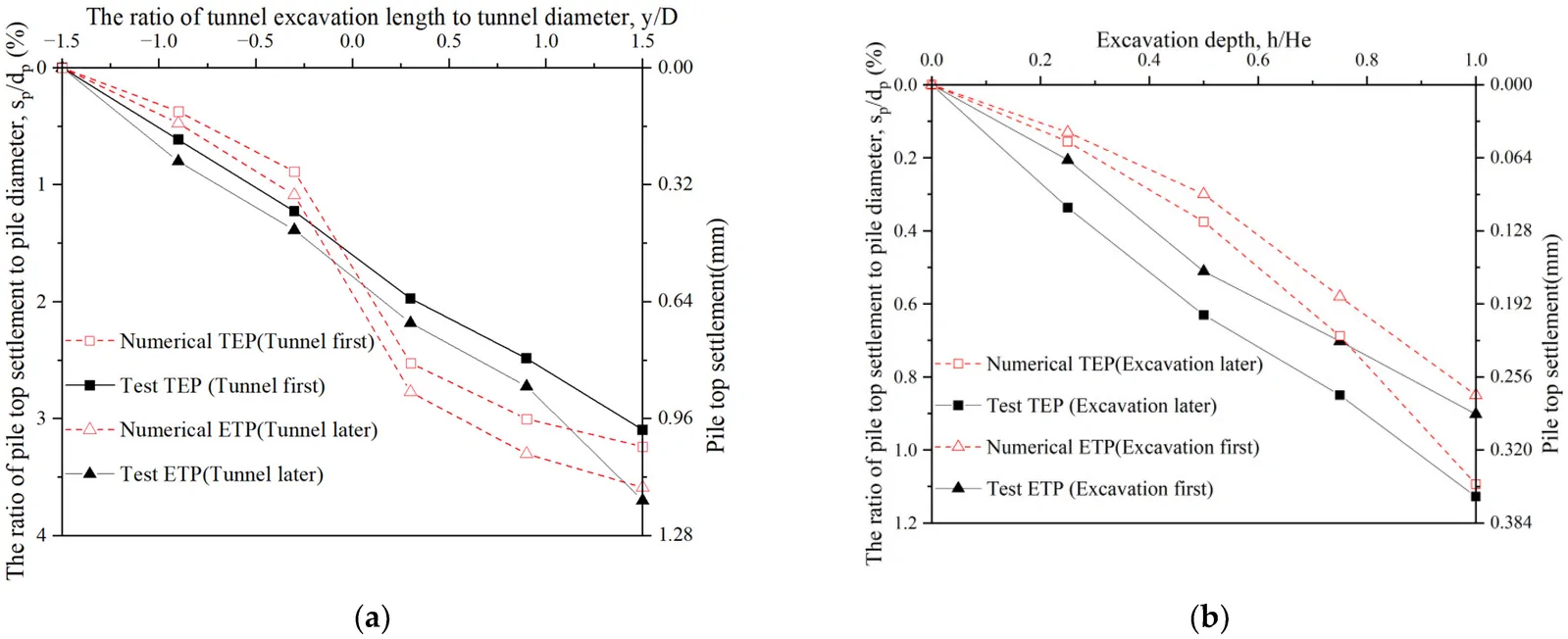
Comparison of numerical and test results of single-pile top settlement: (**a**) tunnel only; (**b**) excavation only.

Figure 24 presents a comparison between the numerical and experimental results of single-pile top settlement for the TEP and ETP cases. As depicted in Figure 24a, the final pile top settlement induced by tunnel excavation was found to be consistent with the model tests. However, a more pronounced increase in settlement was observed during the tunnel passage beneath the pile compared to the experimental results. Despite this discrepancy, the overall trend remained similar, with settlement accumulating continuously as tunnel excavation progressed. Figure 24b indicates that with progressive excavation, the pile top settlement in the numerical model exhibited an accelerating growth rate, whereas the settlement increment per stage in the experiments remained relatively uniform. Nevertheless, the final settlement magnitudes were found to be comparable. This discrepancy may be attributed to the adoption of a simplified, idealized elastoplastic pile–soil interface model in the numerical simulation, which fails to fully capture the complexity of the actual pile–soil interaction.

**Figure 25:**
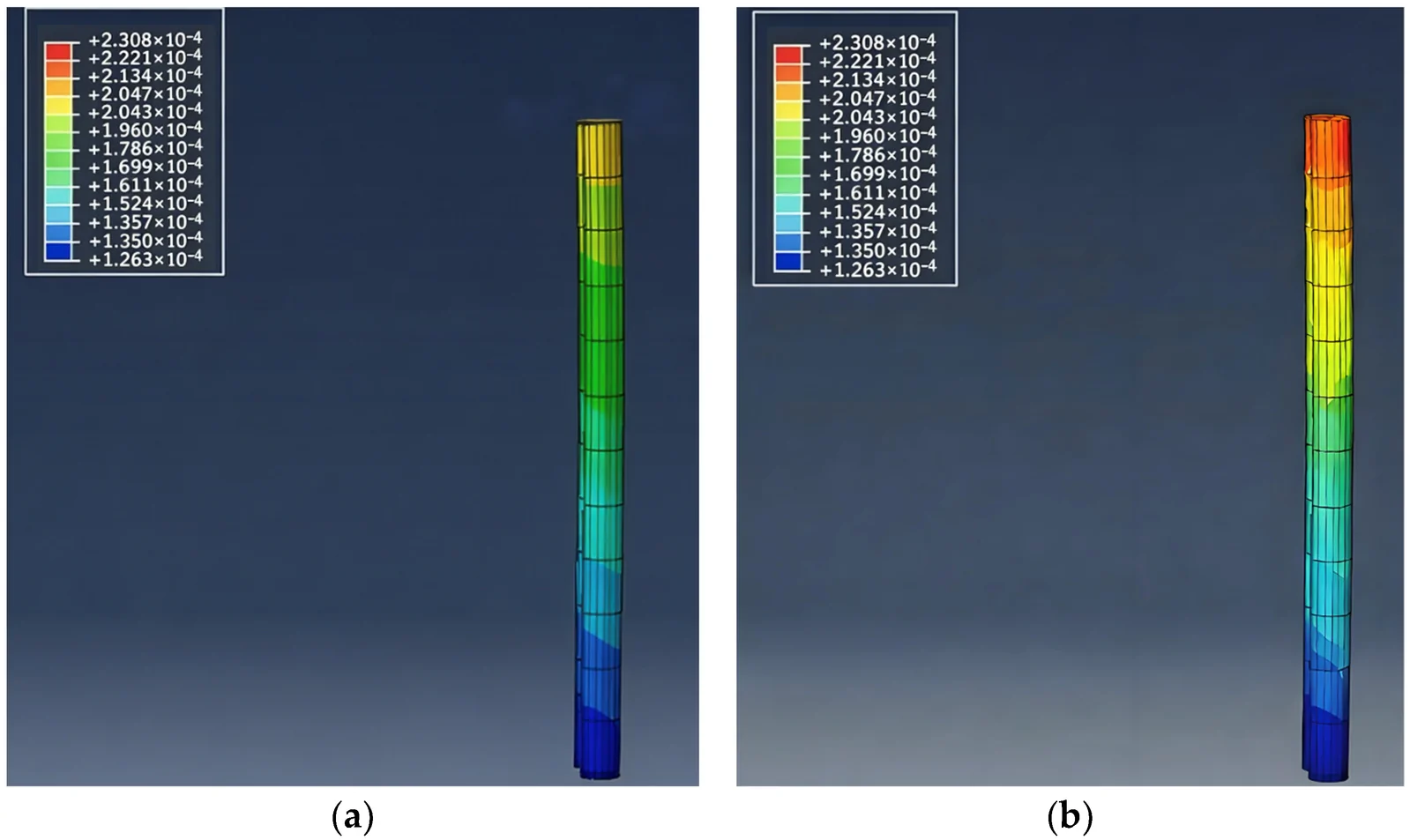
The vertical displacement contour plots of the model pile: (**a**) TEP; (**b**) ETP.

Figure 25 displays contour plots of vertical displacement for the pile foundation under TEP and ETP conditions. The cumulative pile settlement induced by ETP exceeds that from TEP, which agrees with the findings presented in Figure 14 and Figure 24.

#### 4.4.3. Axial Force of Pile Body

**Figure 26:**
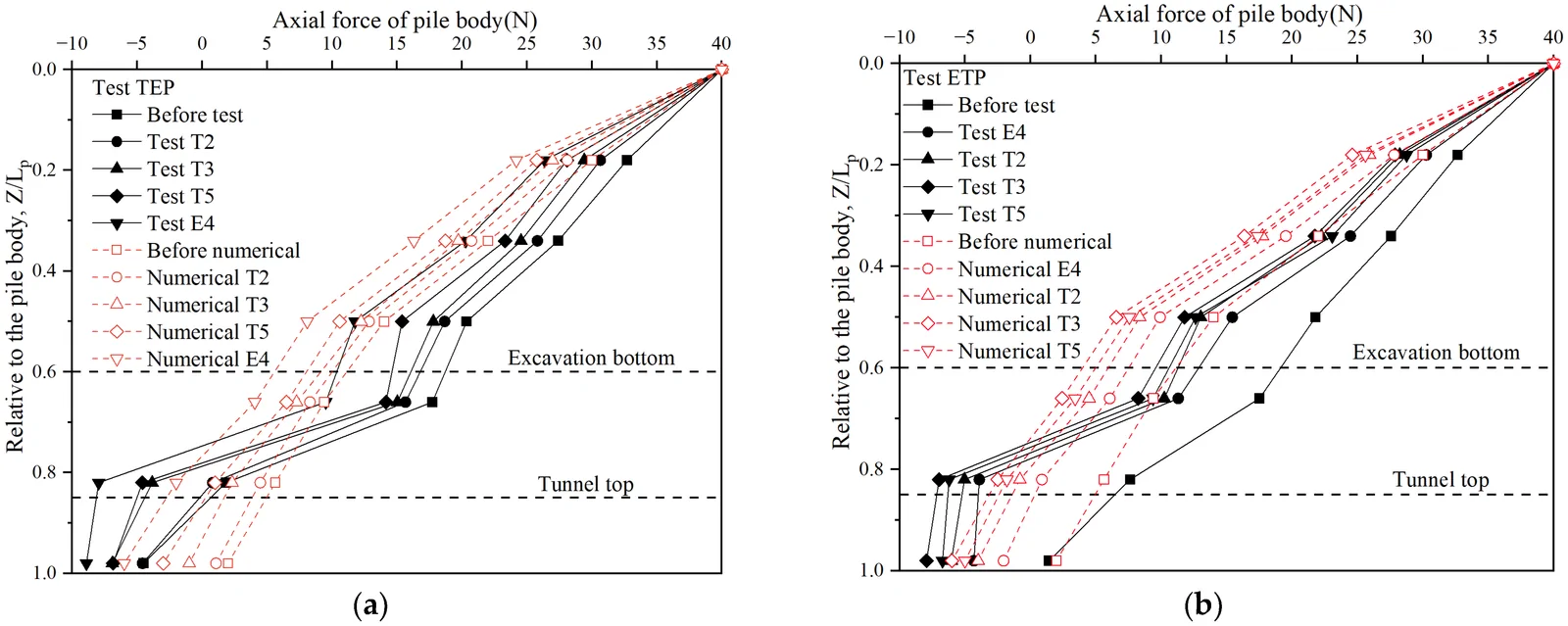
Comparison between numerical value of single-pile axial force and test results: (**a**) TEP; (**b**) ETP.

Figure 26 illustrates the comparison of single-pile axial forces between numerical simulations and model tests. The results reveal a generally consistent variation pattern: the axial force attenuates progressively with depth, with negative axial force developing near the pile tip. For the TEP case, a reduction in pile axial force was induced by both tunnel and excavation activities. In contrast, for the ETP case, the axial force decreased upon completion of excavation. During subsequent tunnel excavation, a continuous decline was observed in stages T2 and T3, followed by a slight recovery in stage T5.

#### 4.4.4. Pile Bending Moment

**Figure 27:**
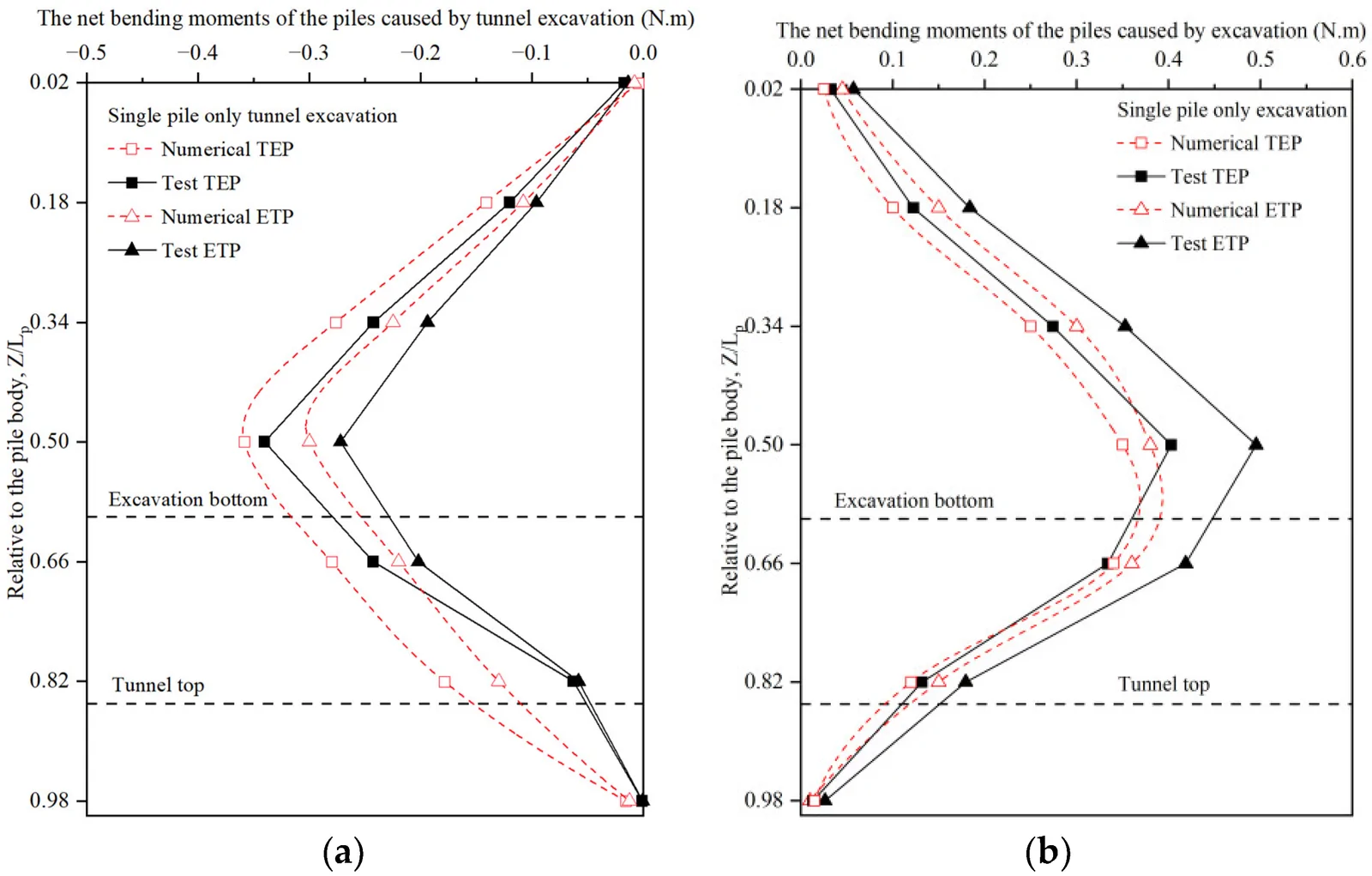
Comparison between numerical and experimental results of bending moment of single-pile shaft: (**a**) tunnel only; (**b**) excavation only.

Figure 27 presents a comparison of the bending moments for a single pile between numerical simulations and model tests. Consistent with previous definitions, tension on the pile surface facing the tunnel is defined as positive bending moment, while compression is defined as negative. As depicted in the figure, the variation patterns of the pile bending moment in the numerical simulation are consistent with the experimental results. Significant bending moments were observed in the region between 0.50 *L_p_* and the excavation base. During tunnel excavation, the negative bending moments were overestimated in the numerical model, whereas during pit excavation, the positive bending moments were underestimated.

Parametric sensitivity analyses reveal that the pile bending moment is most influenced by the small-strain shear modulus and the unloading/reloading modulus of the clay, as these parameters control soil stiffness and lateral displacement under unloading conditions. The at-rest earth pressure coefficient and the pile–soil interface factor also exert measurable influences. These parameters can be conveniently modified within the numerical model to conduct parametric investigations.

To quantitatively assess the agreement between indoor model test data and 3D finite element predictions, the mean relative error and maximum relative error were employed as quantitative metrics for pile top settlement, pile bending moment, ground surface settlement, and excess pore water pressure. Statistical analyses indicate that the mean relative error across all key indices remains below 7.2%, while the maximum relative error does not surpass 12.5%, confirming acceptable computational accuracy for geotechnical analysis.

The deviations between experimental and numerical curves in Figure 22, Figure 23, Figure 24, Figure 25, Figure 26 and Figure 27 are examined as follows: The finite element results tend to slightly underestimate the measured deformation and internal forces. These minor deviations arise primarily from three aspects: (1) the small-strain hardening constitutive model employed in the numerical simulation simplifies the inherent structure and long-term rheological characteristics of natural Hangzhou clay; (2) the clay in the indoor model displays heterogeneity caused by layered compaction, whereas the soil mass in the numerical model is treated as a homogeneous continuum; (3) minor systematic errors are inherent in test sensors and drainage pipelines during physical modeling, which cannot be replicated in an idealized numerical simulation. Nonetheless, the numerical curves capture the overall evolutionary trends well with reasonable error magnitudes, thereby confirming the reliability of the established 3D numerical model.

## 5. Conclusions

(1) The surface settlement profile triggered by tunnel excavation conforms to a typical normal distribution with a “V” shape. The peak settlement occurs at the tunnel centerline, while the minimum is found at −2D from the centerline. The transverse influence zone extends to at least ±2D, and the excavation sequence has a comparatively minor effect on surface settlement. Surface settlement caused by excavation displays an arch-shoulder pattern, reaching its maximum near the outer edge of the support structure and attenuating with distance. TEP and ETP show negligible differences in their influence on surface settlement.
(2) Pile top settlement increases significantly during stage T3 when the tunnel crosses the pile section. Construction sequence has minimal influence on ground settlement (only a 9% difference), yet pile responses vary considerably: ETP produces 9% greater cumulative pile top settlement and 31% higher peak bending moment compared to TEP. Both sequences generate negative excess pore water pressure, though its distribution and dissipation rates differ depending on the unloading order. Overall, the TEP scheme induces less disturbance to the adjacent pile. The final settlement is 4.22% *d_p_* for TEP and 4.60% *d_p_* for ETP. TEP is recommended for engineering practice.
(3) The construction sequence affects the evolution of single-pile axial force. In the TEP case, both construction stages lead to a decrease in pile axial force. In the ETP case, the axial force reduction pattern during the excavation stage remains similar; however, subsequent tunnelling reduces axial force in stages T1–T3 and increases it in stages T4–T5.
(4) Tunnel excavation generates negative bending moments on the tunnel-side pile surface owing to lateral compression, whereas excavation produces positive bending moments due to lateral tension. The peak bending moments occur near 0.50 *L_p_* of the pile shaft. The maximum cumulative bending moment of TEP is 31% lower than that of ETP, verifying the advantage of the TEP scheme.
(5) Combined tunnelling and excavation typically generates negative excess pore water pressure in the surrounding clay, which gradually dissipates over time. Both the magnitude of impact and the induced pressure increase as the distance to the tunnel face or excavation bottom decreases.
(6) Due to the slow dissipation of excavation- and tunnelling-induced excess pore water pressure in clay and its coupling with time-dependent soil deformation, continuous in situ monitoring of pore water pressure, alongside settlement and pile displacement, is recommended throughout the construction period and the subsequent post-construction phase to safeguard adjacent pile foundations.

## Figures and Tables

**Table 1:** Scaled model test program.

Test	Pile Foundation Type	Remark
L	P	Reference static load test for ultimate bearing capacity.
TEP	P	Combined unloading test: tunnel excavation followed by basement excavation.
ETP	P	Combined unloading test: basement excavation followed by tunnel excavation (sequence comparison).

**Table 2:** Soil material parameters.

Parameter	Unit	Clay	Sandy Soil
Constitutive model	—	HSS	HSS
Drainage type	—	Undrainage A	drain
Saturation density (γ_sat_)	kN/m^3^	18.1	20
Triaxial drainage test secant modulus (E_50_^ref^)	kPa	1600	6300
Tangential modulus of principal loading in consolidation test (E_oed_^ref^)	kPa	1300	6300
Unload/Reload Modulus (E_ur_^ref^)	kPa	6200	37,800
Unload/Reload Poisson Ratio (v_ur_)	—	0.2	0.2
Stiffness stress-related power value (m)	—	0.7	0.5
Cohesion (c)	kPa	1	1
Internal friction angle (φ′)	°	15	35
Small strain reference shear modulus (G_0_^ref^)	kPa	17,000	90,000
Shear strain level (γ_0.7_)	—	0.00022	0.0001
Permeability coefficient x-direction (k_x_)	m/day	0.001	0.1
Permeability coefficient y-direction (k_y_)	m/day	0.001	0.1
Permeability coefficient z-direction (k_z_)	m/day	0.001	0.1
Interface reduction factor (R_inter_)	—	0.6	0.5
Initial lateral pressure coefficient (K_0_)	—	automatic	automatic
Constitutive model	—	HSS	HSS

## Data Availability

The original contributions presented in this study are included in the article. Further inquiries can be directed to the corresponding author.
